# Cell Entry of Avian Reovirus Modulated by Cell-Surface Annexin A2 and Adhesion G Protein-Coupled Receptor Latrophilin-2 Triggers Src and p38 MAPK Signaling Enhancing Caveolin-1- and Dynamin 2-Dependent Endocytosis

**DOI:** 10.1128/spectrum.00009-23

**Published:** 2023-04-25

**Authors:** Wei-Ru Huang, Yi-Ying Wu, Tsai-Ling Liao, Brent L. Nielsen, Hung-Jen Liu

**Affiliations:** a Institute of Molecular Biology, National Chung Hsing University, Taichung, Taiwan; b iEGG and Animal Biotechnology Center, National Chung Hsing University, Taichung, Taiwan; c Rong Hsing Research Center for Translational Medicine, National Chung Hsing University, Taichung, Taiwan; d Ph.D Program in Translational Medicine, National Chung Hsing University, Taichung, Taiwan; e Department of Medical Research, Taichung Veterans General Hospital, Taichung, Taiwan; f Department of Microbiology and Molecular Biology, Brigham Young University, Provo, Utah, USA; g Department of Life Sciences, National Chung Hsing University, Taichung, Taiwan; Institute of Molecular Biology, Academia Sinica

**Keywords:** avian reovirus, σC, Src, p38 MAPK, annexin A2, ADGRL2, Csk-Cbp interaction, caveolin-1, dynamin 2

## Abstract

The specifics of cell receptor-modulated avian reovirus (ARV) entry remain unknown. By using a viral overlay protein-binding assay (VOPBA) and an in-gel digestion coupled with liquid chromatography-tandem mass spectrometry (LC-MS/MS), we determined that cell-surface annexin A2 (AnxA2) and adhesion G protein-coupled receptor Latrophilin-2 (ADGRL2) modulate ARV entry. Direct interaction between the ARV σC protein and AnxA2 and ADGRL2 in Vero and DF-1 cells was demonstrated *in situ* by proximity ligation assays. By using short hairpin RNAs (shRNAs) to silence the endogenous AnxA2 and ADGRL2 genes, ARV entry could be efficiently blocked. A significant decrease in virus yields and the intracellular specific signal for σC protein was observed in Vero cells preincubated with the specific AnxA2 and ADGRL2 monoclonal antibodies, indicating that AnxA2 and ADGRL2 are involved in modulating ARV entry. Furthermore, we found that cells pretreated with the AnxA2/S100A10 heterotetramer (A2t) inhibitor A2ti-1 suppressed ARV-mediated activation of Src and p38 mitogen-activated protein kinase (MAPK), demonstrating that Src and p38 MAPK serve as downstream molecules of cell-surface AnxA2 signaling. Our results reveal that suppression of cell-surface AnxA2 with the A2ti-1 inhibitor increased Csk-Cbp interaction, suggesting that ARV entry suppresses Cbp-mediated relocation of Csk to the membrane, thereby activating Src. Furthermore, reciprocal coimmunoprecipitation assays revealed that σC can interact with signaling molecules, lipid raft, and vimentin. The current study provides novel insights into cell-surface AnxA2- and ADGRL2-modulated cell entry of ARV which triggers Src and p38 MAPK signaling to enhance caveolin-1-, dynamin 2-, and lipid raft-dependent endocytosis.

**IMPORTANCE** By analyzing results from VOPBA and LC-MS/MS, we have determined that cell-surface AnxA2 and ADGRL2 modulate ARV entry. After ARV binding to receptors, Src and p38 MAPK signaling were triggered and, in turn, increased the phosphorylation of caveolin-1 (Tyr14) and upregulated dynamin 2 expression to facilitate caveolin-1–mediated and dynamin 2-dependent endocytosis. In this work, we demonstrated that ARV triggers Src activation by impeding Cbp-mediated relocation of Csk to the membrane in the early stages of the life cycle. This work provides better insight into cell-surface AnxA2 and ADGRL2, which upregulate Src and p38MAPK signaling pathways to enhance ARV entry and productive infection.

## INTRODUCTION

Avian reoviruses (ARVs) are important pathogens of birds that have been associated with several diseases in poultry including enteric and respiratory diseases, hepatitis, myocarditis, and malabsorption syndrome (1, 2). ARV is an oncolytic virus (3–6) containing 10 double-stranded RNA genome segments which are classified into three sizes, including three segments of the L-class (L1 to L3), three of the M-class (M1 to M3), and four of the S-class (S1 to S4) that encode at least eight structural and four nonstructural proteins (7). The S1 genome segment contains three open reading frames which are translated into p10, p17, and σC proteins, respectively. ARV σC protein is a cell attachment protein (8) which is analogous to mammalian reovirus (MRV) σ1 attachment protein and possesses both type- and broadly specific epitopes (9). The C-terminal fragment of σC protein (residues 151 to 326) of ARV has been demonstrated to be the receptor-binding globular domain (10). The σC protein is an apoptosis inducer (11) which activates p53 to induce apoptotic signaling, causing transcriptional induction of Bax, and the tyrosine kinase Src functions upstream of p53 (12–14). ARV triggers cytochrome c and Smac/DIABLO release from mitochondria to the cytosol, which induces apoptosis (13). Our previous report suggested that African green monkey kidney (Vero) and immortalized chicken embryo fibroblast (DF-1) cell entry of ARV occurs through caveolin-1–mediated and dynamin- and lipid raft-dependent endocytic pathways that require activation of p38 MAPK and Src signaling pathways (15). Caveolae are a subset of membrane lipid rafts that contain caveolin proteins, which serve as organizing centers for cellular signal transduction (16). A number of signaling molecules interact with the binding motif of caveolin-1 scaffolding domain (16). To date, the cell receptors and the mechanisms underlying signaling pathway-modulated virus entry remain largely unknown.

AnxA2 is a calcium-sensing, phospholipid-binding protein that forms a heterotetramer with its binding partner S100A10. The AnxA2/S100A10 heterotetramer complex consists of two copies of AnxA2 and one copy of the S100A10 dimer. AnxA2 is known to associate with the plasma membrane and the endosomal system. AnxA2 has been identified as a raft-associated protein and preferentially binds to cholesterol and phosphatidylinositol 4,5-bisphosphate–rich domains of lipid rafts (17, 18). The AnxA2-S100A10 complex has been described as an important receptor for tissue-type plasminogen activator (tPA) in endothelium and other cell types (19). On the endothelial cell surface, tPA binds with the AnxA2 complex, facilitating the activation of the main fibrinolytic protease, plasmin (20). Cell-surface AnxA2-generated plasmin appears to not only clear fibrin, but also promote proteolysis of the extracellular matrix (ECM). AnxA2 is generated by different cell types, such as epithelial cells, trophoblasts, neurons, dendrites, tumor cells, monocytes, and macrophages (21–23). In addition to maintaining cell surface proteolytic activity, AnxA2 also fulfills a range of intracellular functions, including membrane repair, endocytosis, exocytosis, and maintenance of adhesion-like intercellular junctions (24). Its functions contribute to fibrinolysis, tissue injury and repair, and regulation of inflammation and immune system activation. The adhesion G protein-coupled receptor Latrophilin-2 (ADGRL2) has been identified as a novel determinant of endothelial cell adhesion and barrier function (25). It has been demonstrated that ADGRL2 inhibits vascular permeability by differential control of endothelial cell adhesion (25). The endogenous ADGRL2 localizes at ECM contacts and inhibits focal adhesion formation and nuclear localization of YAP/TAZ transcriptional regulators, while promoting tight junction assembly (25). Blood vessels are hyperpermeable, and intravascularly injected cancer cells extravasate more easily in *lphn2a* null animals. Thus, ADGRL2 ligands such as FLRT2 may be therapeutically exploited to interfere with cancer metastatic dissemination. Recent reports have suggested that Latrophilin-2 is a novel cell-surface marker for the cardiomyogenic lineage and has functional significance in heart development, suggesting that the cardiomyogenic marker ADGRL2 can be used to facilitate targeted stem cell therapy for heart repair in clinical applications (25).

The aim of this work was to perform a comprehensive study to identify proteins on the host cell surface which modulate cell entry and to elucidate the mechanism underlying ARV entry-triggered signaling pathways for enhancing ARV entry. This work reveals for the first time that cell-surface AnxA2 and ADGRL2 modulate cell entry of ARV, which triggers Src and p38 MAPK signaling during or post-entry to increase phosphorylation of caveolin-1 (Tyr14) and expression of dynamin 2, thereby facilitating ARV entry into host cells.

## RESULTS

### Identification of ARV σC protein binding to cell-surface proteins by VOPBA and LC-MS/MS.

**FIG 1:**
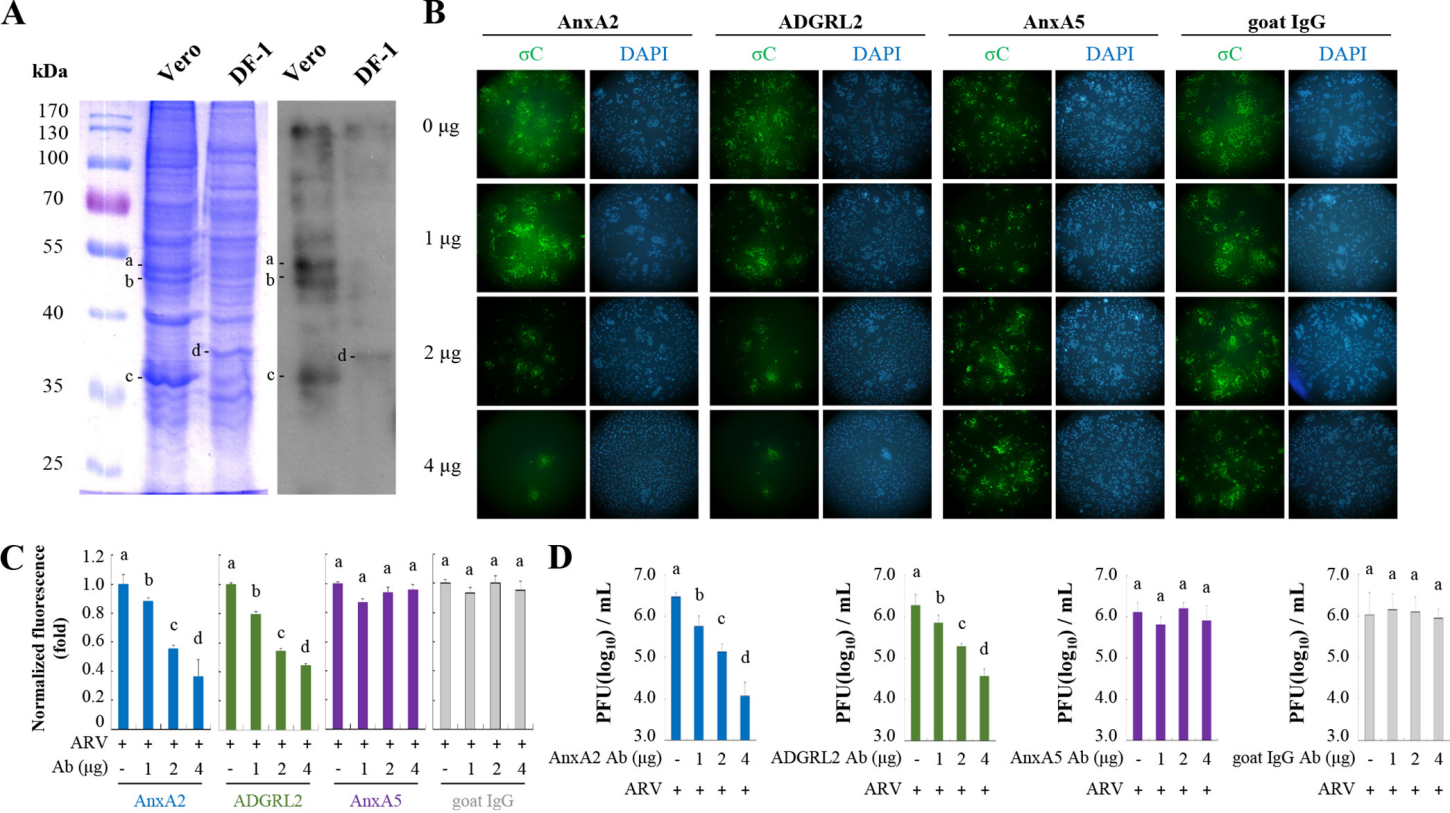
Viral overlay protein-binding assay (VOPBA) analysis of avian reovirus (ARV) binding to Vero and DF-1 cell membrane proteins. (A) SDS-PAGE shows the Vero and DF-1 cell membrane proteins in the left panel. VOPBA was used to identify membrane proteins that bind to ARV as shown in the right panel. Binding of ARV to membrane proteins is shown at approximately 32 and 50 kDa, indicated by bands a, b, c, and d. Band a, ADGRL2; band b, AnxA5; bands c and d, AnxA2. These proteins were further identified by liquid chromatography-tandem mass spectrometry (LC-MS/MS) analysis. (B) Detection of internalized σC protein in ARV by antibody blocking assays. Confluent monolayers of Vero cells, grown in 8-well chambers for 24 h, were pre-incubated or not with anti-AnxA2, -AnxA5, and-ADGRL2 antibodies at the indicated concentrations for 1 h at 37°C. Goat-IgG was used as a negative control. After extensive washing, cells were incubated with ARV at a multiplicity of infection (MOI) of 10 for 1 h, and the cells were fixed and stained with DAPI (4′,6-diamidino-2-phenylindole; blue) and antibodies specific for σC (green). (C) Fluorescence signals in panel B were quantified with ImageJ software. The amount of fluorescence in the mock control group was considered to be 1-fold. Duncan’s multiple range test (MDRT) using SPSS software was used to analyze the statistical significance of all data. (D) Virus yields were determined as treatments from panel B. Significance between treatments was determined by MDRT using SPSS software (version 20.0). Means with common lowercase letters (a, b, c, d) indicate no significant difference at *P* < 0.05. Each value is the mean (with standard error, SE) from three independent experiments.

Membrane proteins of DF-1 and Vero cells were analyzed by SDS-PAGE as shown in Fig. 1A (left panel). To identify the ARV σC protein that binds to cell-surface proteins, we performed a viral overlay protein-binding assay (VOPBA) and in-gel digestion coupled with liquid chromatography-tandem mass spectrometry (LC-MS/MS). The binding of ARV σC to membrane proteins is shown in Fig. 1A (left panel). Of these candidate proteins, four bands migrating at 35 to 55 kDa (Fig. 1A, bands a, b, c, and d) were excised for two-dimensional LC-MS/MS analysis to identify the specific proteins (Fig. 1A, right panel). Bands a and b are ADGRL2 and AnxA5, respectively, while bands c and d are both AnxA2. Among the identified proteins, AnxA2, AnxA5, and ADGRL2 were evaluated (Table 1). With VOPBA, interactions that rely on a certain conformation may be missed due to the denaturing nature of the gel. To evaluate the participation of these proteins in ARV entry, we treated monolayers of Vero cells with 0, 1, 2, and 4 μg of anti-AnxA2, -AnxA5, and -ADGRL2 monoclonal antibodies to block these proteins. After the removal of excess antibodies, cells were incubated with the ARV S1133 strain at a multiplicity of infection (MOI) of 10. Cells were fixed, permeabilized, and immunolabeled to detect internalized ARV σC protein. Internalization of σC (fluorescent signals) was determined and compared in the presence and absence of the antibody. A significant decrease in the intracellular specific signal for the ARV σC protein was observed in Vero cells preincubated with the specific AnxA2 and ADGRL2 monoclonal antibodies (2 and 4 μg) compared to the mock control (Fig. 1B). No significant difference in the signal was observed when the cells were preincubated with 1 μg of the antibody (Fig. 1B). Furthermore, no difference in the signal was observed in cells preincubated with AnxA5 monoclonal antibody or goat IgG antibody (negative control) (Fig. 1B). Analysis of fluorescence signals showed a significant decrease in σC protein levels in cells pretreated with the specific AnxA2 and ADGRL2 antibodies (Fig. 1C). The virus titers in Vero cells treated with AnxA2 and ADGRL2 monoclonal antibodies were 2.5- and 3-log units lower than those of the untreated samples (Fig. 1D). The virus titers in cells preincubated with the AnxA5 monoclonal antibody or goat IgG antibody were not altered compared with those of the untreated group (Fig. 1D).

**TABLE 1:** Identification of ARV-binding membrane proteins by LC-MS/MS^*a*^

Observed	Mr.		Delta	Score	Expected value	Match sequence
	Expected	Calculated				
AnxA2						
366.2176	730.4206	730.4225	−0.0018	54	0.00041	K.ELASALK.S
381.2323	760.4500	760.4517	−0.0017	56	0.00014	K.LMVALAK.G
437.2804	872.5461	872.5517	−0.0056	69	4e-006	R.KLMVALAK.G
440.7244	879.4342	879.4338	0.0004	55	0.0003	R.DLYDAGVK.R
518.7739	1,035.5332	1,035.5349	−0.0017	61	6.4e-005	R.DLYDAGVKR.K
526.2646	1,050.5146	1,050.5168	−0.0022	40	0.0081	K.WISIMTER.S
543.7461	1,085.4776	1,085.4778	−0.0001	62	3.1e-005	K.AYTNFDAER.D
544.3022	1,086.5898	1,086.5921	−0.0022	74	3e-006	R.DALNIETAIK.T
556.2799	1,110.5453	1,110.5458	−0.0005	76	1.7e-006	R.QDIAFAYQR.R
611.7999	1,221.5852	1,221.5877	−0.0025	99	1.1e-008	K.TPAQYDASELK.A
623.3078	1,244.6010	1,244.5997	0.0014	102	4.7e-009	R.TNQELQEINR.V
451.8912	1,352.6518	1,352.6572	−0.0054	63	3.9e-005	K.DIISDTSGDFRK.L
535.5914	1,603.7524	1,603.7552	−0.0028	69	1e-005	K.SYSPYDMLESIRK.E
545.6219	1,633.8440	1,633.8423	0.0016	71	5.5e-006	R.TNQELQEINRVYK.E
547.2712	1,638.7917	1,638.7923	−0.0006	105	2.7e-009	K.TPAQYDASELKASMK.G
556.9406	1,667.8001	1,667.8015	−0.0015	59	0.00011	R.SNAQRQDIAFAYQR.R
615.6418	1,843.9036	1,843.8952	0.0084	119	9.9e-011	K.LSLEGDHSTPPSAYGSVK.A
AnxA5						
501.3004	1,000.5863	1,000.5917	−0.0053	61	3.9e-005	K.VLTEIIASR.T
507.7576	1,013.5006	1,013.5069	−0.0063	32	0.045	R.LYDAYELK.H
400.9010	1,199.6813	1,199.6873	−0.0061	55	0.00014	R.SEIDLLNIRK.E
ADGRL2						
430.7819	859.5492	859.5280	0.0213	26	0.029	K.LQLIAFR.N

a ARV, avian reovirus; LC-MS/MS, liquid chromatography-tandem mass spectrometry; AnxA2, annexin A2; ADGRL2, adhesion G protein-coupled receptor Latrophilin-2.

### The ARV σC protein interacts with cell surface AnxA2 and ADGRL2.

**FIG 2:**
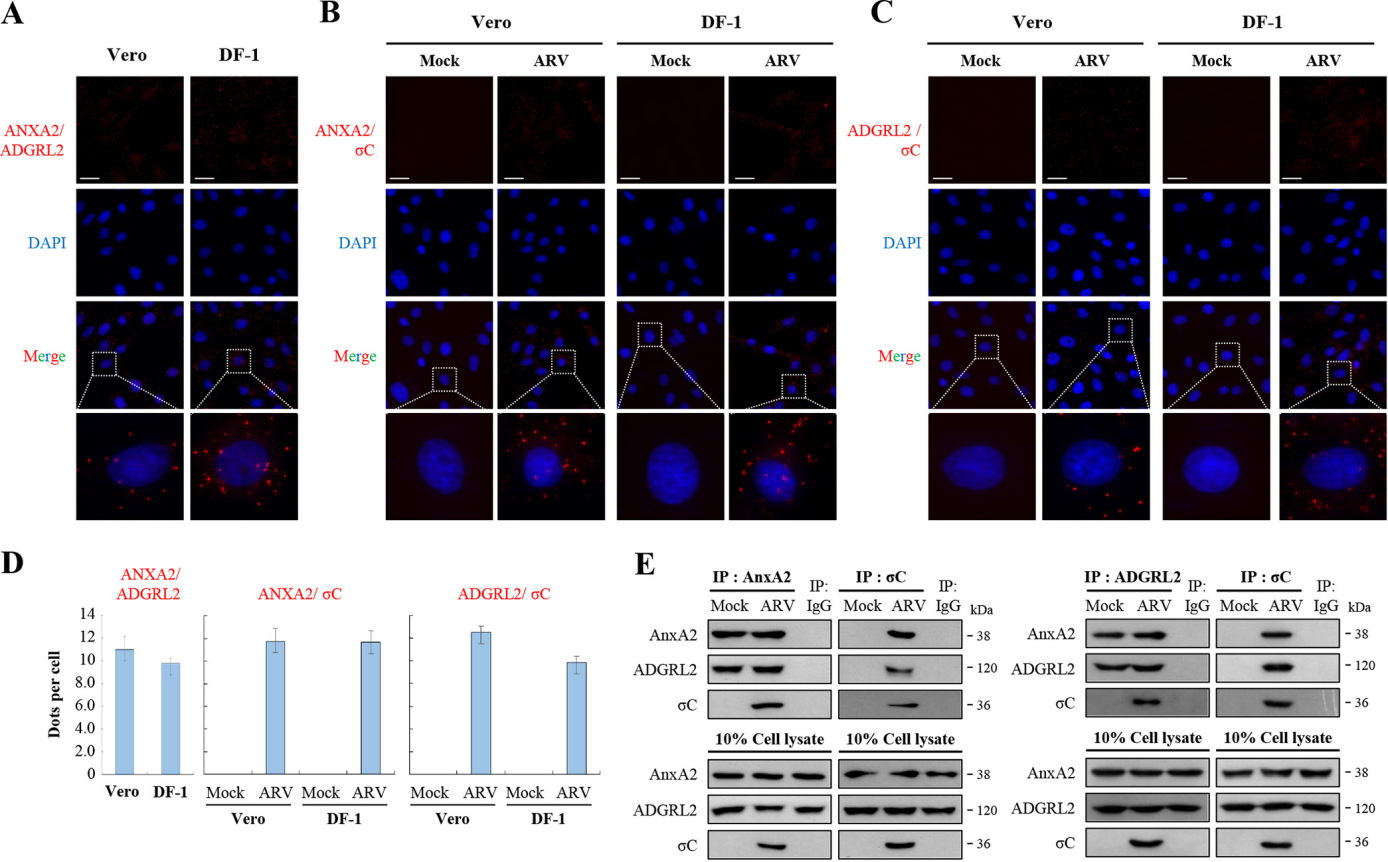
Proximity ligation assays (PLA) for cell-surface AnxA2 and ADGRL2 in Vero and DF-1 cells. (A to C) PLAs for ARV σC protein and cell surface AnxA2 and ADGRL2 receptors in Vero and DF-1 cells. To analyze the interactions of AnxA2/ADGRL2 (A), σC/AnxA2 (B), and σC/ADGRL2 (C), interactions between Vero and DF-1 cells were detected using a Duolink commercial kit (Sigma-Aldrich, cat no. DUO 92008) based on *in situ* PLA based on the manufacturer’s instructions. Enlarged images correspond to the region indicated by the white box in the merged image. Representative images are from three independent experiments. (D) The fluorescence bright spots in Fig. 2A, B, and C were quantified, with each bar graph (mean ± SE) representing the average value obtained from about 100 cells in 5 randomly selected regions. (E) To confirm the interaction of ARV σC protein with AnxA2, ADGRL2 or AnxA2/ADGRL2 in Vero cells, the cell membrane fraction was isolated for reciprocal co-immunoprecipitation assays. IgG was used as a negative control. Predicted size (kDa) of each protein is labeled to the right of gels and blots in each figure. All original/uncropped blots and images from this study are provided in Fig. S8 in the supplemental material.

The proximity ligation assay (PLA) is a new powerful technique to visualize protein-protein interaction (26–28). To determine whether ARV σC protein interacts with cell-surface AnxA2 and ADGRL2 in Vero and DF-1 cells, the interactions between them on Vero and DF-1 cells were analyzed *in situ* by PLA. Earlier reports have shown that PLA can be used to visualize the interaction between two proteins present on the same cells or two different cells (26–28). Our results clearly indicated that cell-surface AnxA2 interacts with ADGRL (Fig. 2A) and that ARV σC protein interacts with cell-surface AnxA2 and ADGRL in Vero and DF-1 cells (Fig. 2B and C). The fluorescence bright spots in Fig. 2A, 2B and 2C were quantified and shown in Fig. 2D. In contrast, no signal was observed in either of the two technical negative controls run in parallel (Fig. 2B and C), showing the specificity of the red dot signals observed. In this work, we also examined colocalization of ARV σC with cell-surface AnxA2 and ADGRL2 as well as annexin A2 and ADGRL2. Our results reveal that colocalization of AnxA2/ADGRL2 (Fig. S1A in the supplemental material), σC/AnxA2 (Fig. S1B), and σC/AnxA2 (Fig. S1C) were observed in ARV-infected Vero and DF-1 cells. Virus titers in ARV-infected Vero and DF-1 cells reached 10^6.1^ and 10^5.5^, respectively, at 24 h postinfection. To further confirm that the ARV σC protein can interact with AnxA2 and ADGRL2 in Vero cells, we isolated the cell membrane fraction for reciprocal co-immunoprecipitation assays. The results revealed that σC co-immunoprecipitated with AnxA2 and ADGRL2 (Fig. 2E). Importantly, we found that ADGRL2 co-immunoprecipitated with AnxA2 (Fig. 2E), suggesting that they are located together in the cell membrane. Collectively, our results reveal that cell-surface AnxA2 and ADGRL2 play critical roles in modulating ARV entry.

### Inhibition of cell-surface AnxA2 and ADGRL2 suppresses virus replication.

**FIG 3:**
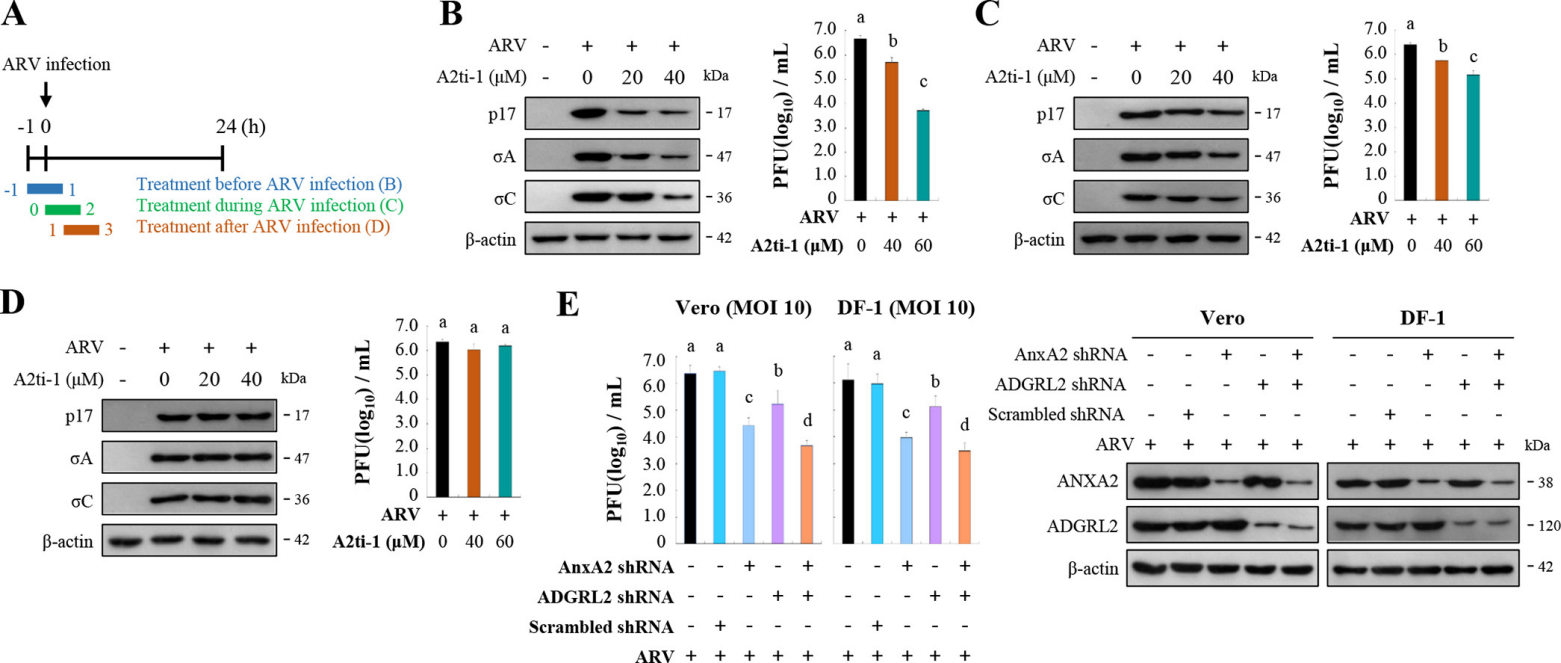
Inhibition of cell-surface AnxA2 and ADGRL2 suppressed virus replication. (A) Experimental design for pulse treatment with the A2ti-1 inhibitor in ARV-infected cells during the early stage of the viral life cycle. (B to D) Vero cells were pretreated with inhibitor A2ti-1 before (B), during (C), and after (D) infection for 2 h. Cells were washed to remove the drug and further incubated with ARV at a MOI of 10 until 24 h. The expression levels of viral proteins p17, σA, and σC were analyzed by Western blotting with the respective antibodies. Virus titers were also determined. All experiments were conducted in triplicate, and data are presented as the means ± SE. (E) Since ADGRL2 inhibitor was not available, we used a short hairpin RNA (shRNA) to deplete the ADGRL2 gene and determined the virus yield. Cells were transfected with the indicated shRNAs for 6 h followed by infection with virus at a MOI of 10 for 24 h. Protein levels were normalized to that of β-actin. Levels of the indicated proteins in the mock treatment were considered 1-fold. Immunoblots in panels B, C, D, and E were quantitated by densitometric analysis using ImageJ software and normalized to that of β-actin. Data are shown in Fig. S3A to D in the supplemental material.

To obtain direct evidence that AnxA2 is involved in the virus life cycle, different concentrations of A2ti-1 were used to inhibit AnxA2 before, during, and after infection. A2ti-1 is a selective and high-affinity AnxA2/S100A10 heterotetramer (A2t) inhibitor and specifically disrupts AnxA2 and S100A10 interaction (29). Figure 3A shows the experimental design for pulse treatment with the A2ti-1 inhibitor in ARV-infected cells during the early stages of the viral life cycle. In this work, we examined protein synthesis alongside virus yield. We pretreated cells with the A2ti-1 inhibitor for 1 h, washed them to remove the drug, and then infected them with ARV at a MOI of 10 for 24 h. Furthermore, to investigate whether A2ti-1 inhibitor and short hairpin RNAs (shRNAs) have deleterious effects on the cell, we assessed the viability of the cells using a 3-(4,5-dimethyl-2-thiazolyl)-2,5-diphenyl-2H-tetrazolium bromide (MTT) assay. Cell viability in A2ti-1–treated and AnxA2- and ADGRL2-knockdown cells was only slightly reduced compared to that in the mock-treatment cells (Fig. S2). Figure 3B and Fig. S3A reveal that the expression levels of viral proteins p17, σA, and σC were reduced in A2ti-1–treated cells. The virus titers in the treated groups were 1- to 2.3-log units lower than that of the untreated sample (Fig. 3B). The expression levels of viral proteins and virus titers of the treated groups during infection were moderately reduced compared to those in the untreated sample (Fig. 3C; Fig. S3B), while the treated groups after infection were not altered (Fig. 3D; Fig. S3C). Because ADGRL2 inhibitor was not available, we used a shRNA to deplete the ADGRL2 gene. As shown in Fig. 3E, virus yield was significantly reduced in both AnxA2- and ADGRL2-knockdown Vero and DF-1 cells compared to that in untreated cells. The expression levels of AnxA2- and ADGRL2-knockdown Vero and DF-1 cells are shown in Fig. 3E (right panel) and Fig. S3D.

### ARV-modulated increased levels of p-Src and p-p38 MAPK enhances phosphorylation of caveolin-1 Tyr14 and expression levels of dynamin 2.

**FIG 4:**
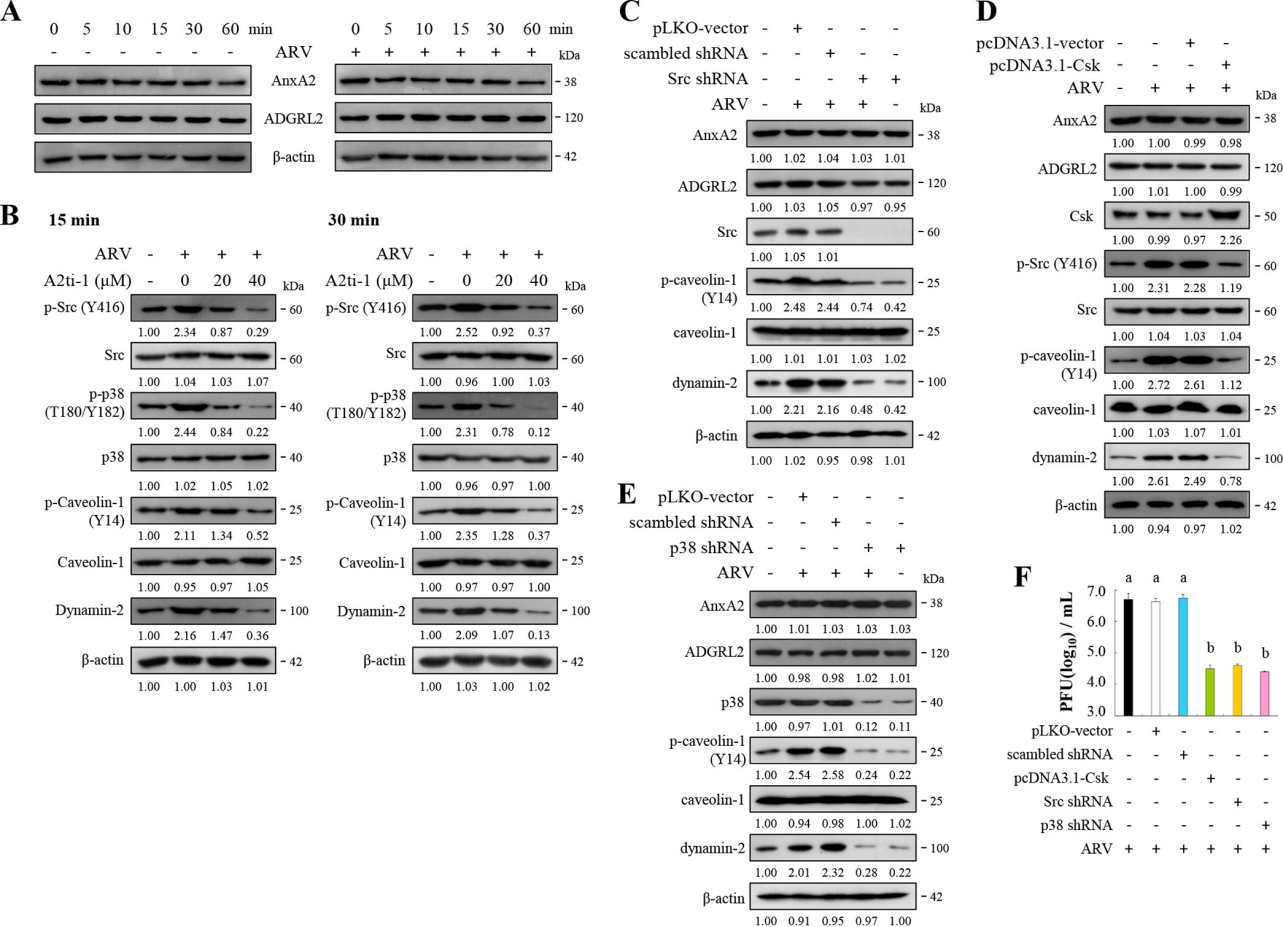
Src and p38MAPK are key downstream molecules of cell-surface AnxA2. (A) Levels of AnxA2 and ADGRL2 were examined in ARV-infected Vero and DF-1 cells at the indicated time points. (B) Vero cells were pretreated with inhibitor A2ti-1 for 1 h and then infected with ARV at a MOI of 10 at the indicated time points. Expression levels of the indicated proteins were analyzed by Western blotting with the respective antibodies, quantitated by densitometric analysis using ImageJ, and normalized to that of actin. The levels of indicated proteins in the mock group are considered 1-fold. The predicted size of each protein (kDa) is labeled to the right of gels and blots in each figure. Numbers below each lane indicate the relative fold of the control level for each specific protein in mock-treated cells. (C to E) Vero cells were transfected with pcDNA3.1-Csk plasmid and shRNAs for 6 h followed by infection with ARV at a MOI of 10 for 24 h. Cell lysates were collected 24 h postinfection and immunoblotted with the respective antibodies. Protein levels were normalized to that of β-actin. The levels of the indicated proteins in the mock treatment are considered 1-fold. Image shown is from a single experiment and is representative of at least three separate experiments. (F) To examine whether the Csk-Src pathway and p38 MAPK regulate virus propagation, we performed overexpression of Csk or knockdown of Src and p38 MAPK. Virus titer was determined. Each value is the mean (with SE) from three independent experiments.

To date, the details of signaling-modulated ARV entry and the cell receptors involved remain largely unknown. Our findings presented in this work inspired us to examine whether inhibition of ARV σC binding to cell-surface AnxA2 blocks activation of Src and p38 MAPK during the early stages of the virus life cycle. We found that the levels of AnxA2 and ADGRL2 were not altered in ARV-infected Vero and DF-1 cells at 60 min postinfection (Fig. 4A; Fig. S4A). To study whether AnxA2 and ADGRL2 play a role in regulating this signaling pathway, we used A2ti-1 inhibitor and shRNAs to suppress AnxA2 and ADGRL2. Our results revealed that before viral infection, inhibition of AnxA2 by A2ti-1 inhibitor suppressed ARV-modulated upregulation of the phosphorylation of Src, p38 MAPK, and caveolin-1 at Y14, as well as the expression levels of dynamin 2, at the 15- and 30-min time points (Fig. 4B; Fig. S4B and C). Interestingly, when ARV-infected cells were treated with A2ti-1 inhibitor (40 μm), a greater reduction in phosphorylation of Src, p38 MAPK, and caveolin-1 was observed compared to the negative controls (Fig. 4B; Fig. S4B and C). To examine whether A2ti-1 can inhibit the Src and p38 MAPK signaling pathways, we treated Vero cells with different concentrations of A2ti-1. As shown in Fig. S4D, we found that A2ti-1 can reduce the levels of phosphorylated Src (p-Src) and p38 MAPK (p-p38 MAPK) in a dose-dependent manner. To further study whether AnxA2 and ADGRL2 play a role in upregulating these signaling pathways, we transfected Vero cells with the respective AnxA2 and ADGRL2 shRNAs for 24 h to knock down AnxA2 and ADGRL2, then infected them with ARV at a MOI of 10 for 30 min. The results revealed that knockdown of AnxA2 reversed the ARV elevation of Src and caveolin 1 phosphorylation at Y14 as well as the increased dynamin 2 level, but further enhanced viral upregulation of p38 MAPK, which is consistent with a previous report (30) (Fig. S4E). Knockdown of ADGRL2 reversed ARV upregulation of Src, p38 MAPK, and caveolin 1 phosphorylation at Y14 and the elevated dynamin 2 level (Fig. S4F).

To mitigate the possibilities of off-target effects or the inhibitor concentrations used being insufficient to affect ARV, we undertook carboxyl-terminal Src kinase (Csk) overexpression and shRNA knockdown experiments targeting Src and p38 MAPK, respectively. The results revealed that suppression of Src and p38 MAPK decreases ARV-modulated caveolin 1 phosphorylation at Y14 and dynamin 2 expression levels (Fig. 4C to E; Fig. S5A to C). The protein levels of cell-surface AnxA2 and ADGRL2 were not altered in Src and p38 MAPK-knockdown cells (Fig. 4C to E; Fig. S5A to C), suggesting that Src and p38 MAPK do not regulate cell-surface AnxA2 and ADGRL2. As shown in Fig. 4F, the virus titer was 2.5-log units lower than those of the untreated or mock-treated groups. Collectively, we demonstrated that ARV σC interacting with cell-surface AnxA2 and ADGRL2 triggers signaling pathways to elevate phosphorylation of caveolin 1 Tyr14 and dynamin 2expression, thereby enhancing virus entry.

### σC protein is associated with Flot-2, vimentin, Src, and Ras.

Our previous study suggested that Src and p38 MAPK molecules activated by ARV are associated with caveolin-1 and dynamin-2 (15). This inspired us to further examine whether σC interacts with Flot-2 (lipid marker), vimentin, Src, Ras, and p38 MAPK. Our results revealed that immunoprecipitation of σC using σC monoclonal antibodies and Western blotting with vimentin, Flot-2, Src, p38 MAPK, and Ras antibodies revealed that these proteins are associated (Fig. S6A to D). In reciprocal experiments in which cell lysates were immunoprecipitated with vimentin, Flot-2, Src, and Ras antibody, σC was detected by Western blotting (Fig. S6A to D). We found that σC did not associate with p38 MAPK (Fig. S6E). In an attempt to detect the phosphorylated Src and p38 MAPK, however, we only detected unphosphorylated total proteins and not the phosphorylated forms under our experimental conditions.

### Inhibition of lipid rafts, Src, and p38 MAPK signaling blocks ARV entry and subsequent infection.

**FIG 5:**
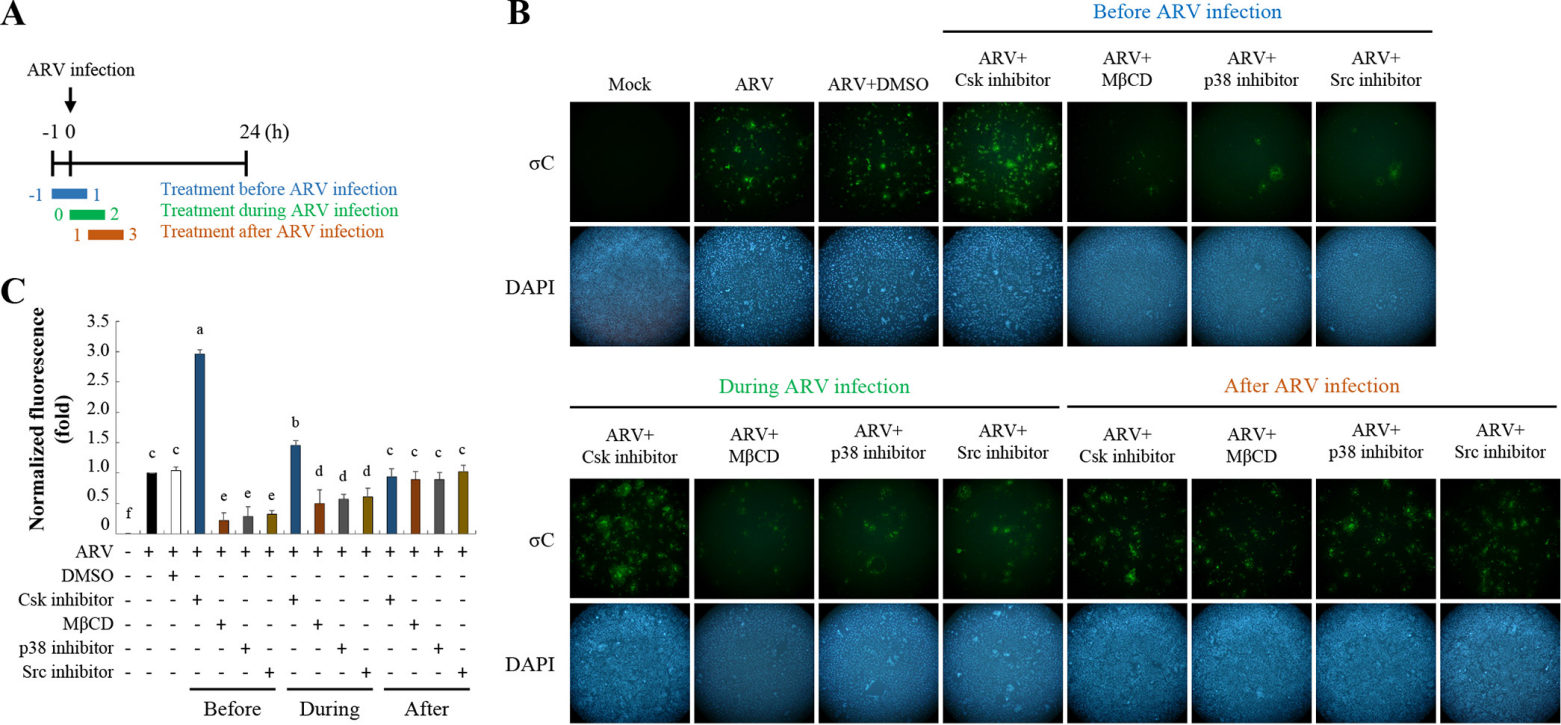
Suppression of lipid raft, Src, and p38 MAPK inhibits ARV entry. (A) Experimental design for pulse treatment with inhibitors. (B) Vero cells were treated with Csk inhibitor (5 μM), MβCD (3.2 mM), p38 MAPK inhibitor (5 μM), and Src inhibitor (5 μM) during different time windows before, during, and after ARV infection at a MOI of 10. The cells were washed to remove the drug and further incubated for 24 h. Immunofluorescence signals were detected at 24 h postinfection using a monoclonal antibody against σC protein and observed by fluorescence microscopy to visualize viral protein expression (green). Cell nuclei were stained with DAPI (blue). (C) Fluorescence signals in panel B were quantified with ImageJ software. The amount of fluorescence in the mock control group was considered to be 1-fold. MDRT was used to analyze the statistical significance of all data.

To further confirm whether lipid rafts (Flot-2) and signaling molecules (Src and p38 MAPK) play important roles in ARV entry, cells were pretreated with different inhibitors before, during, and after ARV absorption for 2h. In this work, 3-IB-PP1, PP2, SB202090, and methyl-β-cyclodextrin (MβCD) inhibitors were used to suppress signaling molecules (Csk, Src, and p38 MAPK) and cholesterol-dependent and raft/caveola-mediated endocytosis (15). Figure 5A shows the experimental design for administering these inhibitors to ARV-infected cells at different time points during the viral life cycle. Vero cells were treated with the indicated inhibitors during different time windows before and after ARV infection at a MOI of 10 for 24 h. The cells were washed to remove the drug and further incubated until 24 h postinfection. All drug-treated and untreated cells were collected 24 h postinfection for immunostaining. When the respective inhibitors were added during or before the adsorption period, the σC protein expression levels detected by immunostaining greatly decreased (Fig. 5B); however, only a limited effect was observed during the post-entry stage (Fig. 5B). Importantly, σC protein expression levels were increased in Csk inhibitor-treated Vero cells before the ARV adsorption period (Fig. 5B). These results suggested that Src and p38 MAPK signaling are important for the early stage of ARV infection. Several studies have suggested that MβCD, which disrupts the cholesterol-rich microdomain, results in the inhibition of both caveolin-dependent endocytosis and caveola-independent, lipid raft-dependent endocytosis (16). In this work, we found that the σC protein expression levels detected by immunostaining were dramatically reduced in MβCD-treated Vero cells. This result is consistent with that of our previous study demonstrating that the cholesterol present in host cytoplasmic membranes is important for early stages of ARV infection (15), further suggesting that lipid rafts play important roles in ARV entry. Analysis of fluorescence signals showed significant decreases or increases in σC protein levels with the respective treatments before infection (Fig. 5C). Taken together, our results revealed that lipid raft and Src and p38 MAPK signaling play critical roles in ARV entry.

### ARV decreases Csk binding to Cbp in the early stage of its life cycle.

**FIG 6:**
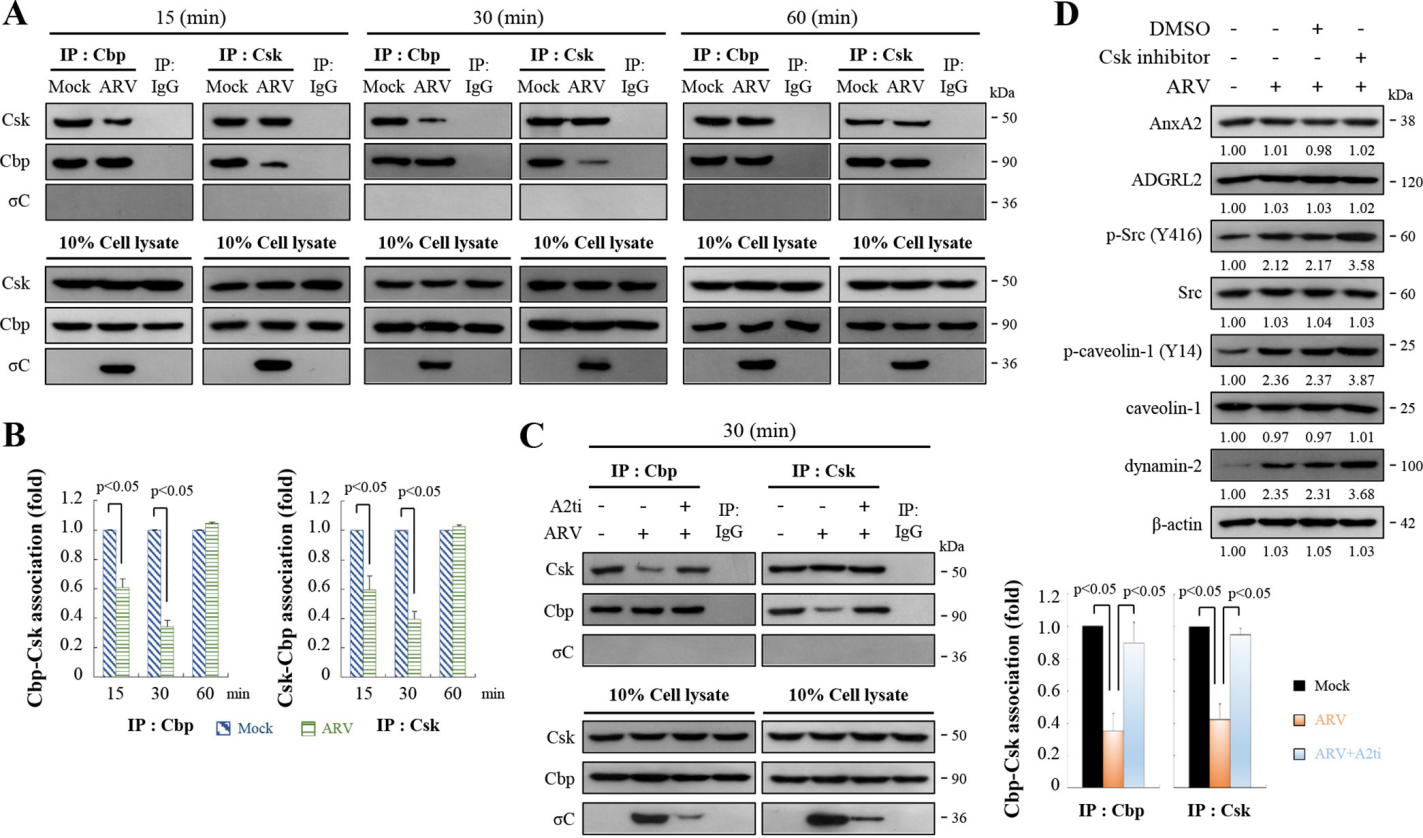
ARV reduces Cbp-Csk interaction during the early stage of its life cycle. (A) Vero cells were infected with ARV at a MOI of 10. Cell lysates were collected at the indicated time points. In co-immunoprecipitation experiments, cells lysates were immunoprecipitated with Cbp or Csk antibodies and analyzed by Western blotting assays with the indicated antibodies. (B) Densitometry analysis results for Western blotting represent the amount of protein association in panel B. Mock-treated cells were considered 1-fold. Signals for all blots were quantified using Image J software. All experiments were conducted independently in triplicate. Student’s *t* test was used to analyze the statistical significance of all data. (C) Left panel: Vero cells were pretreated with inhibitor A2ti-1 before infection for 2 h. Cells were washed to remove the drug and further incubated with ARV at a MOI of 10 for 30 min. Cell lysates were immunoprecipitated with Cbp or Csk antibodies and analyzed by Western blotting assays with the indicated antibodies. Right panel: Western blots (left panel) were quantitated by densitometric analysis using ImageJ and normalized to β-actin. Right panel is expressed as folds representing the amount of protein and protein association. Mock cells were considered 1-fold. Each value is the mean (with SE) from three independent experiments. Student’s *t* test was used to analyze the statistical significance of all data. (D) Vero cells were pretreated with CSK inhibitor before infection for 2 h. Cells were washed to remove the drug and further incubated with ARV at a MOI of 10 for 24 h. The expression levels of the indicated proteins were analyzed by Western blotting with the respective antibodies. Image shown is from a single experiment and is representative of at least three separate experiments.

Csk is a cytoplasmic protein and therefore requires the adaptor Cbp for recruitment to the plasma membrane to phosphorylate a critical tyrosine residue in each of the Src family kinases (SFKs), which suppresses their activities (31–33). The transmembrane protein Cbp can recruit Csk to the membrane where the SFKs are located. To investigate whether ARV activates Src at 15 to 30 min postinfection (Fig. 4B) by suppressing Cbp-mediated relocation of Csk to the membrane, we examined the amount of Csk-Cbp interaction at 15-, 30-, and 60-min postinfection. As shown in Fig. 6A to B, ARV reduced the amount of Csk binding to Cbp at 15- to 30-min postinfection during the early stage of the life cycle. In addition, interactions of σC with Csk or Cbp were not observed in reciprocal co-immunoprecipitation assays (Fig. 6A). Previous studies have suggested that Cbp-mediated relocation of Csk to the membrane plays a role in turning off the signaling events initiated by SFKs (31–33). Immunoprecipitation results suggested that ARV-modulated reduction of Csk-Cbp association was reversed in A2ti-1–pretreated cells (Fig. 6C, right and left panels), suggesting that ARV binding to cell-surface AnxA2 is important for suppressing Cbp-mediated relocation of Csk to the membrane, thereby activating Src. Furthermore, the levels of p-Src, p-p38 MAPK, p-caveolin-1 Tyr14, and dynamin 2 in ARV-infected cells were further increased in cells which were pretreated with CSK inhibitor before infection for 2 h (Fig. 6D; Fig. S7). The expression levels of cell-surface AnxA2 and ADGRL2 were not altered in CSK inhibitor-treated cells (Fig. 6D; Fig. S7). Our results reveal that ARV entry suppresses Cbp-mediated relocation of Csk to the membrane, thereby activating Src to upregulate the phosphorylated form of caveolin 1 Tyr14 and dynamin 2 expression during the early stage of the life cycle.

## DISCUSSION

**FIG 7:**
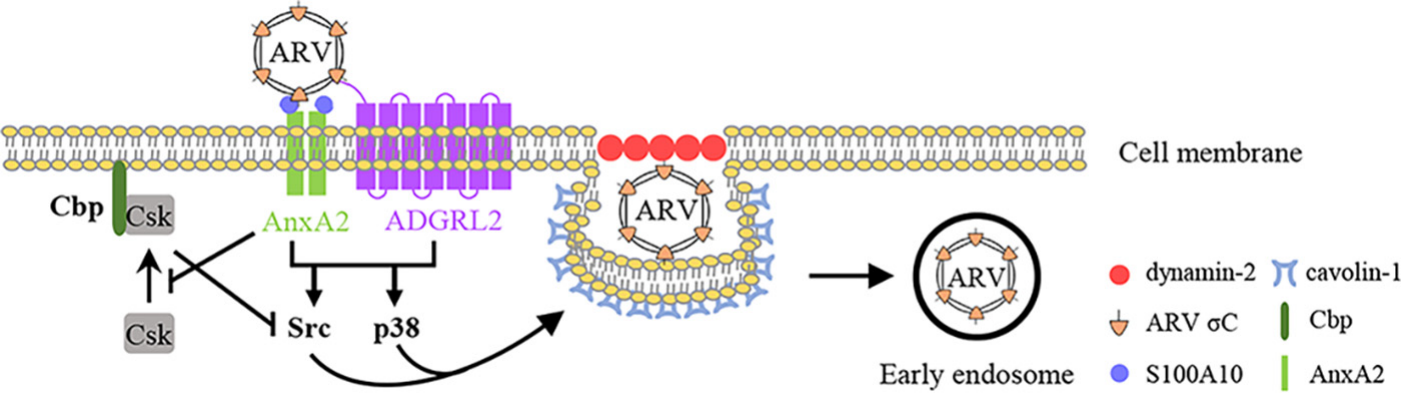
Schematic diagram showing the binding of ARV to cell-surface AnxA2 and ADGRL2, which activates cellular signaling pathways to enhance caveolin-1- and dynamin 2-dependent endocytosis. This study indicates that AnxA2 and ADGRL2 mediate cell entry of ARV. ARV σC protein binding to cell-surface AnxA2 and ADGRL2 of Vero and DF-1 cells activates Src and p38 MAPK signaling to increase caveolin-1 phosphorylation and upregulate dynamin 2 expression, thereby facilitating ARV entry through caveolin-1-, dynamin 2-, and lipid-raft-dependent endocytosis. Importantly, ARV activates Src by impeding Cbp-mediated relocation of Csk to the membrane during the early stage of its life cycle. Arrows (→) indicate activation; bars (⊥) indicate repression.

The ARV life cycle is highly dependent on cellular factors for viral entry, replication, assembly, and virion release (4, 14, 34–39). Although ARV entry into cells occurs through caveolin-1- and dynamin 2-dependent endocytic pathways (15), the virus receptors and cell receptor-modulated signaling pathways which facilitate ARV entry remain unknown. Using VOPBA and LC-MS/MS, we identified cell-surface AnxA2 and ADGRL2 as cell-surface binding receptors for ARV entry. A schematic diagram illustrating the mechanisms of AnxA2- and ADGRL2-modulated signaling pathways to promote ARV entry is shown in Fig. 7.

Several lines of evidence were obtained which prove that AnxA2 and ADGRL2 modulate ARV entry: (i) only those cells expressing the cell-surface AnxA2 and ADGRL2 were able to internalize σC; (ii) the entry of σC was affected by anti-AnxA2 and -ADGRL2 antibodies; (iii) σC internalization was reduced in Vero and DF-1 cells silenced for cell-surface AnxA2 and ADGRL2 expression; (iv) and interaction between σC and cell-surface AnxA2 and ADGRL2 was observed *in situ*. To our knowledge, this is the first report to reveal that cell-surface AnxA2 and ADGRL2 modulate ARV entry and that ARV entry activates Src and p38 MAPK signaling pathways, which increase caveolin-1 phosphorylation and upregulate dynamin 2 expression during the early stage of the virus life cycle, thereby promoting virus entry and productive infection.

Previous reports have suggested that ADGRL2 is a marker for heart development and induces myocardial repair after infarction (25, 40). In contrast, AnxA2 is used by many viruses as a cellular receptor. Several enveloped and nonenveloped viruses have been shown to utilize AnxA2 or A2t to some capacity during their life cycle (22, 41, 42). AnxA2 or A2t has been implicated in the attachment and entry of several viruses (22, 41, 42). There have been several reports of AnxA2-virus associations occurring within cell types other than epithelial cells (22, 23). Interestingly, we found that the AnxA2/S100A10 heterotetramer mediates ARV entry into both Vero and DF-1 cells. Previous studies have suggested that several human cancer lines are selectively infected by ARV (3–6, 43). ARV may utilize different cellular receptors for entry into different cancer cell lines. It is possible that ARV uses different receptors and entry pathways *in vivo* in chickens than those it uses in DF-1 cells. Moreover, ARVs infect many different organs, and different strains have different preferred tissue tropisms. ARV may use different receptor binding and entry pathways in different tissues. Further experiments deciphering the mechanisms underlying the tropism of ARV in bird tissues and ARV entry into different cancer cell lines are needed. A deeper understanding of how AnxA2 biology is manipulated during the cycle of viral infections may uncover novel treatment routes or expand our understanding of viral pathogenesis.

Viral infections are known to activate various cellular signaling pathways to facilitate their entry and replication (44–46). An increasing amount of information has shown that several viruses activate the Src and p38 MAPK pathways to augment efficient replication (12, 46, 47). To this end, although our group has demonstrated that Src and p38 MAPK activation is beneficial for ARV entry, apoptosis induction, and replication (12, 14, 15, 47), little is about known about the underlying mechanisms. Our group previously reported that ARV-modulated activation of Src and p38 MAPK signaling pathways is beneficial for ARV entry (15). We obtained several lines of evidence proving that ARV activates Src and p38 MAPK signaling. First, using the cell-surface AnxA2 inhibitor, we demonstrated that Src and p38 MAPK are key for downstream signaling of cell-surface AnxA2. Inhibition of AnxA2 dramatically reduced phosphorylation of Src and p38 MAPK. In this work, we further demonstrated that suppression of σC interaction with cell-surface AnxA2 and ADGRL2 receptors by an inhibitor or specific monoclonal antibodies impedes activation of Src and p38 MAPK and ARV entry, suggesting that virus receptor binding is required to activate Src and p38 MAPK signaling. The C-terminal fragment of σC protein (residues 151 to 326) is the receptor-binding globular domain (10). Although this fragment has the same topology as the head domain of the MRV σ1 cell attachment protein, the virus receptor for σ1 protein is the junctional adhesion molecule-A (48), which is different from ARV receptors. Further studies deciphering the structural basis of σC with its receptor/co-receptor interactions and the underlying mechanisms which trigger cellular signaling are now in progress. Furthermore, our findings reveal that Src and p38 MAPK play critical roles in regulating caveolin-1 phosphorylation and dynamin 2 expression to assist virus entry. Second, in this work we explored whether ARV regulates Csk recruitment to the plasma membrane to bind Cbp. Csk, a non-receptor tyrosine kinase, serves as an indispensable negative regulator of Src family kinases (31–33). Importantly, our findings revealed that ARV activates Src by suppressing Csk-Cbp interaction during the early stage of the virus life cycle, suggesting that ARV impedes Csk recruitment to the plasma membrane to inactivate Src.

Because MβCD, which disrupts the cholesterol-rich microdomain, inhibits both caveolin-dependent endocytosis and caveola-independent, lipid raft-dependent endocytosis (34, 35), we found that inhibition of cholesterol-rich lipid rafts by MβCD dramatically inhibited ARV entry, indicating that lipid rafts play an important role in modulating ARV entry. Several reports have suggested that the cholesterol-rich lipid rafts are involved in different stages of the life cycle in many enveloped and even nonenveloped viruses (49–51). Parton et al. (52) suggested that cholesterol plays an important role in the formation of caveolae and that the N-terminal end of the caveolin scaffolding domain of caveolin-1 contains a conserved serine residue which mediates cholesterol binding. This study suggests the importance of lipid rafts for ARV entry. In conclusion, our findings demonstrate that cell-surface AnxA2 and ADGRL2 mediate cell entry of ARV. ARV has evolved to trigger multiple signaling pathways to promote its entry and the formation of endocytic vesicles during the early stage of its life cycle.

## MATERIALS AND METHODS

### Cell lines, reagents, and antibodies.

We used the attenuated vaccine strain of ARV, S1133, in this study. Vero and DF-1 cells were cultured in Dulbecco’s modified Eagle’s medium (DMEM) supplemented with 5% fetal bovine serum (FBS), 1% penicillin-streptomycin, and 10 mM HEPES (pH 7.2) at 37°C in a 5% CO_2_ incubator. One day prior to each experiment, all tested cells were seeded in 6-cm cell culture dishes with 1× 10^6^ cells in a 37°C incubator with 5% CO_2_. To determine whether AnxA2 was involved in the virus life cycle, we used a selective, high-affinity A2t inhibitor, A2ti-1. MβCD, PP2 (Src inhibitor), 3-IB-PP1 (Csk inhibitor), and SB202190 (p38 MAPK inhibitor) were purchased from Merck (Darmstadt, Germany). The p17 polyclonal antibodies and the σA and σC monoclonal antibodies were produced by our laboratory. Rabbit anti-annexin V and anti-LPHN2 were obtained from Abcam (Cambridge, United Kingdom). Rabbit anti-AnxA2, rabbit anti-p-caveolin-1 (Y14), rabbit anti-caveolin-1, rabbit anti-dynamin I/II, rabbit anti-p-Src (Y416), rabbit anti-Src, rabbit anti-p-p38 (T180/Y182) MAPK, rabbit anti-p38 MAPK, rabbit anti-vimentin, rabbit anti-flotillin-2, and rabbit anti-Ras antibodies were purchased from Cell Signaling (Danvers, MA, USA). Mouse anti-β-actin antibody was obtained from Millipore (Billerica, MA, USA). Mouse anti-Csk and anti-Cbp antibodies were obtained from Santa Cruz Biotechnology (Dallas, TX, USA). Goat anti-mouse IgG (H+L) HRP (horseradish peroxidase), goat anti-rabbit IgG (H+L) HRP, goat anti-mouse IgG FITC (fluorescein isothiocyanate)-labeled, Alexa Fluor 488, and goat anti-rabbit IgG TRITC (tetramethyl rhodamine isocyanate)-labeled Alexa Fluor 546 antibodies were purchased from SeraCare (Milford, MA, USA). Peroxidase-conjugated Affinipure goat anti-rabbit IgG Fc fragment-specific secondary antibodies were purchased from Jackson ImmunoResearch Laboratories Inc. (West Grove, PA, USA).

### shRNAs used in this study.

The pLKO-AS1-puro plasmid-encoding shRNAs were obtained from the National RNAi Core Facility, Academia Sinica, Taiwan. The target sequences for Src, p38 MAPK, AnxA2, and ADGRL2 are as follows: 5′-GACAGACCTGT CCTTCAAGAA-3′ (cat no. TRCN0000038150), 5′-CAAAGTTC GAGTAGCT ATC AA-3′ (TRCN0000010040), 5′-CGGGATGCTTTGAACATTGAA-3′ (TRCN000 0056 145), and 5′-GCCAATGAACTGGCTAAACAT-3′ (TRCN0000011726), respectively. Vero or DF-1 cells were transfected with the respective shRNA for 6 h followed by infection with ARV at a MOI of 10 for 24 h, respectively. Whole-cell lysates were collected for Western blotting.

### Viral overlay protein binding assay.

To identify the related receptors for ARV entry, the viral overlay protein binding assay was used to identify which receptors on the cell membrane bind to the virus. The membrane proteins from Vero or DF-1 cells were isolated using CNMCS Compartmental Protein Extraction kit (BioChain, Newark, CA, USA) according to the manufacturer’s protocol. Briefly, the prepared membrane proteins were separated by 10% SDS-PAGE and transferred to a polyvinylidene fluoride membrane (GE Healthcare Life Sciences, Chicago, IL, USA). The membrane containing membrane proteins was blocked with SUPERBLOCK T20 solution (Thermo Fisher, Waltham, MA, USA) at room temperature for 1 h. After blocking, the membrane was incubated with ARV in PBST overnight at 4°C. Subsequently, the membrane was washed three times with PBST buffer and incubated with the anti-σC monoclonal antibody overnight at 4°C. After washing thrice with PBST, the membrane was incubated with HRP-conjugated anti-mouse IgG antibody for 2 h at room temperature. The target proteins were detected on X-ray films after incubation of the membrane with enhanced chemiluminescence solution. Another identical gel was stained with Coomassie brilliant blue. The positions of the major viral binding bands were isolated from duplicated gels and sent for mass spectrometry (LC-MS/MS) analysis.

### Antibody blocking assay.

Confluent monolayers of Vero, grown in 8-well chambers for 24 h, were pre-incubated or not with anti-AnxA2, AnxA5, and-ADGRL2 antibodies at concentrations of 1, 2, 4 μg/mL for 1 h at 37°C. After extensive washing, cells were incubated with ARV at a MOI of 10 for 1 h, and the cells fixed and processed for detection and quantification of the internalized σC protein of ARV.

### Plasmid construction.

The pcDNA3.1-Csk and pCI-neo-σC constructs were described previously (12, 38).

### Isolation of the plasma membrane fraction.

To investigate interaction of σC with AnxA2 and ADGRL2 as well as interaction of AnxA2 and ADGRL2 in the cytoplasm membrane, the plasma membrane fraction of Vero cells was isolated using a CNM compartmental protein extraction kit according to the manufacturer’s protocol (BioChain Inc., Hayward, USA). Briefly, cells were seeded into 6-cm cell culture dishes. At about 75% confluence, the cells were infected with ARV at a MOI of 10. All cultures were harvested at 24 h postinfection for coimmunoprecipitation assays.

### Coimmunoprecipitation assays.

To investigate the interaction of σC-cellular receptor, σC-signaling molecules, and Csk-Cbp, immunoprecipitation was performed using the Catch and Release kit (Upstate Biotechnology) based on the manufacturer’s protocol. The detailed procedures were described previously (37). Vero cells were seeded in 6-cm cell culture dishes with DMEM containing 5% FBS and incubated at 37°C with 5% CO_2_ until cell confluence reached about 75%. Cells were infected with ARV at a MOI of 10 or transfected with the respective plasmid DNA and collected at 15 min, 30 min, 60 min or 24 h postinfection or posttransfection. Cells were washed twice with phosphate-buffered saline (PBS) and lysed in 300 mL of 3-[(3-cholamidopropyl)-dimethylammonio]-1-propanesulfonate (CHAPS) lysis buffer (40 mM HEPES [pH 7.5], 1 mM EDTA, 10 mM glycerophosphate, 120 mM NaCl, 50 mM NaF, 10 mM pyrophosphate, and 0.3% CHAPS). One thousand micrograms of total proteins collected from each sample was incubated with 2 mg of the respective indicated antibodies, or rabbit IgG (negative control) at 4°C for 24 h. The immunoprecipitated proteins were separated by SDS-PAGE followed by Western blotting with the respective antibodies. Rabbit IgG was used as negative control.

### Proximity ligation assays.

Interactions between AnxA2/ADGRL2, ARV σC/AnxA2, and ARV σC/ADGRL2 Vero and DF-1 cells were detected using the commercial kit Duolink (Sigma-Aldrich, cat no. DUO 92008) based on an *in situ* proximity ligation assay (PLA) used following the manufacturer’s instructions. PLA allows the detection of direct protein-protein interactions at distances of <40 nm in intact fixed cells (26–28). Briefly, Vero or DF-1 cell monolayers were grown on 8-well chambers, preincubated with ARV at a MOI of 100, and, after 30 min of incubation, fixed with 4 % paraformaldehyde for 10 min at room temperature. After incubation with a blocking buffer for 30 min at 37°C, cells were incubated with the respective antibodies as primary antibodies. After several washes, cells were incubated with the PLA probes linked, anti-mouse, and anti-rabbit secondary antibodies. Finally, the cells were subjected to ligation and amplification reactions. The PLA signal was detected in a confocal microscope (Inverted Olympus MPhot) using a ×60 immersion oil objective and values were determined using the mean of three images per condition in three independent experiments. Image analysis was carried out using Fiji ImageJ software.

### Immunofluorescence staining.

To examine of the internalized σC protein of ARV by antibody blocking assays, confluent monolayers of Vero cells, grown in 8-well chambers for 24 h, were pre-incubated or not with anti-AnxA2, AnxA5, and-ADGRL2 antibodies at the indicated concentrations for 1 h at 37°C. To colocalize AnxA2/ADGRL2, σC/AnxA2, and σC/ADGRL2, Vero and DF-1 cells were infected with ARV at a MOI of 10 for 24 h. Co-localization of AnxA2 and ADGRL2 and σC/AnxA2 in cells stained with DAPI (4′,6-diamidino-2-phenylindole), and antibodies specific for AnxA2, ADGRL2, and σC. To study whether lipid rafts and signaling molecules (Src and p38 MAPK) play important roles in ARV entry, 3-IB-PP1 (5 μM), PP2 (5 μM), SB202090 (5 μM), and MβCD (3.2 mM) inhibitors were used to suppress Csk, Src, and p38 MAPK and to block cholesterol-dependent and raft/caveola-mediated endocytosis (15). Vero cells were pretreated with different inhibitors before, during, and after ARV absorption for 2 h. Cells were washed to remove the drug and further incubated until 24 h. Cells were fixed with 4% paraformaldehyde (Alfa Aesar, Haverhill, MA, USA) for 20 min at room temperature and processed to detect internalized ARV σC protein. Fixed cells were incubated in PBS with 0.1% Triton X-100 for 10 min. Cells were washed twice with 1× PBS and blocked with SUPERBLOCK T20 solution (Thermo Scientific, Waltham, MA, USA) at room temperature for 1 h. After two washes with 1× PBS, cells were incubated with the indicated primary antibodies at 4°C overnight. The solution was removed and cells were washed three times in PBS, 5 min each wash. Cells were incubated with FITC-conjugated secondary antibody (Alexa Fluor 488, green) at 4°C overnight (in the dark). Cell nuclei were stained with DAPI for 10 min. After three washes with 1× PBS, cells were observed under a fluorescence microscope. Fluorescence signals were quantified with ImageJ software.

### Electrophoresis and Western blotting assays.

Cells were seeded in 6-well cell culture dishes 1 day before infection with virus or transfection with the respective indicated plasmid DNA. Collected samples were washed twice with 1× PBS and lysed with lysis buffer. The concentrations of solubilized protein in the cell lysates were determined with the Bio-Rad protein assay (Bio-Rad Laboratories, Hercules, CA, USA) according to the manufacturer’s protocol. Equal amounts of samples were mixed with 2.5× Laemmli loading buffer and boiled for 10 min in a water bath. The samples were electrophoresed in a 10% or 12% SDS-polyacrylamide gel and transferred to a polyvinylidene difluoride membrane (GE Healthcare Life Sciences, Chicago, IL, USA). Protein expression levels were analyzed using the appropriate primary antibodies and horseradish peroxidase secondary antibody conjugates. The results were detected on X-ray films (Kodak, Rochester, NY, USA) after membrane incubation with the enhanced chemiluminescence reagent (Amersham Biosciences, Little Chalfont, United Kingdom).

### Determination of virus titer.

To determine the effects of virus receptors and signaling molecules on ARV entry and virus replication, Vero or DF-1 cells were treated with an AnxA2 inhibitor or transfected with the respective shRNAs for 6 h followed by infection with the ARV S1133 strain at a MOI of 10 for 24 h, respectively. Collected cell samples were destroyed by freezing and thawing three times. The supernatant was centrifuged at 12,000 × *g* for 10 min at 4°C to collect the intracellular fraction and stored at −80°C for further virus titration. Virus titers were determined by an agar-covered plaque assay performed in triplicate as described previously (37).

### Statistical analysis.

Duncan’s multiple range test using SPSS software (version 20.0) or Student’s *t* test was used to analyze the statistical significance of all data obtained in this study. Each value represents the mean ± standard error of three independent experiments.
