# Supplementary material for: Cell Entry of Avian Reovirus Modulated by Cell-Surface Annexin A2 and Adhesion G Protein-Coupled Receptor Latrophilin-2 Triggers Src and p38 MAPK Signaling Enhancing Caveolin-1- and Dynamin 2-Dependent Endocytosis
Source: Microbiol Spectr. 2023 Apr 25;11(3):e00009-23. doi: 10.1128/spectrum.00009-23 (PMC10269738; doi:10.1128/spectrum.00009-23)
Supplement: Supplemental file 1 — Supplemental material. Download spectrum.00009-23-s0001.pdf, PDF file, 12.8 MB [file spectrum.00009-23-s0001.pdf]

Supplementary information

Cell entry of avian reovirus modulated by cell-surface annexin A2 and adhesion G protein-coupled receptor Latrophilin 2 triggers Src and p38 MAPK signaling enhancing caveolin 1- and dynamin 2-dependent endocytosis

Wei-Ru Huang[a,b], Yi-Ying Wu[a, b], Tsai-Ling Liao[c,d,e], Brent L. Nielsen[f], Hung-Jen Liu[a, b,c,d,g*]

[a]Institute of Molecular Biology, National Chung Hsing University, Taichung, Taiwan

[b]The iEGG and Animal Biotechnology Center, National Chung Hsing University, Taichung, Taiwan

[c]Rong Hsing Research Center for Translational Medicine, National Chung Hsing University, Taichung, Taiwan

[d]Ph.D Programin Translational Medicine, National Chung Hsing University, Taichung, Taiwan

[e]Department of Medical Research, Taichung Veterans General Hospital, Taichung, Taiwan

[f]Department of Microbiology and Molecular Biology, Brigham Young University, Provo, Utah, USA

[g]Department of Life Sciences, National Chung Hsing University, Taichung, Taiwan

Corresponding author and address reprint requests:

Dr. Hung-Jen Liu, Institute of Molecular Biology, National Chung Hsing University, Taichung, Taiwan

Address: 145 Xingda Rd., South Dist., Taichung City 402, Taiwan

Tel:886-4-22840485 ext. 243; Fax: 886-4-22874879

E-mail: hjliu5257@nchu.edu.tw.

Table S1. The catalog numbers and dilution factor of the respective antibodies used in this study

Antibodies	Catalog numbers	Clone name	Dilution factor	Manufacture
*Mouse anti-p17	-	-	2000	Our laboratory
Mouse anti-σA	-	-	4000	Our laboratory
Mouse anti-σC	-	-	4000	Our laboratory
Rabbit anti-Annexin V	ab108194	EPR3980	2000	abcam
*Rabbit anti-LPHN2	ab139498	-	2000	abcam
Rabbit anti-Annexin A2	8235	D11G2	2000	Cell Signaling
Rabbit anti-p-Caveolin-1 (Y14)	3251	-	2000	Cell Signaling
Rabbit anti-Caveolin-1	3238	-	2000	Cell Signaling
*Rabbit anti-Dynamin I/II	2342	-	2000	Cell Signaling
Rabbit anti-p-Src (Y416)	6943	D49G4	1000	Cell Signaling
Rabbit anti-Src	2123	32G6	2000	Cell Signaling
*Rabbit anti-p-p38 (T180/Y182)	9211	-	2000	Cell Signaling
Rabbit anti-p38 MAPK	8690	D13E1	2000	Cell Signaling
Rabbit anti-Vimentin	5741	D21H3	2000	Cell Signaling
Rabbit anti-Flotillin-2	3436	C42A3	2000	Cell Signaling
Rabbit anti-Ras	3339	27H5	2000	Cell Signaling
Mouse anti-Csk	sc-166560	E-3	1000	Santa Cruz
Mouse anti-Cbp	sc-365387	G-8	1000	Santa Cruz
Mouse anti-β-actin	MAB1501	C4	10000	Millipore
Goat anti-mouse IgG (H+L) HRP	5220-0341	-	5000	SeraCare
Goat anti-rabbit IgG (H+L) HRP	5220-0336	-	5000	SeraCare
Goat anti-mouse IgG (H+L) FITC-Labeled, Alexa Fluor® 488	5230-0307	-	500	SeraCare

*Polyclonal antibodies

Figure S1

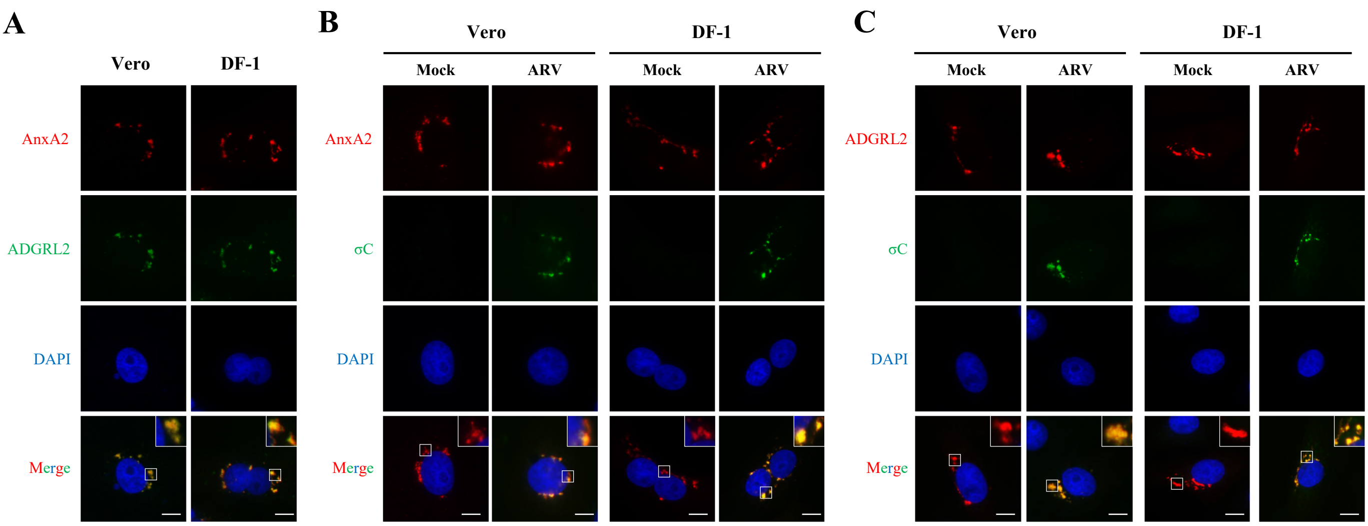

Supplementary Fig. S1. Cell surface AnxA2 and σC/ADGRL2 receptors are involved in ARV entry. (A-C) To colocalize AnxA2/ADGRL2 (A), σC/AnxA2 (B), and σC/ADGRL2 (C), Vero and DF-1 cells were infected with ARV at MOI of 10 for 24 hours. Co-localization of AnxA2 and ADGRL2 and σC/AnxA2 in cells stained with DAPI (blue), and antibodies specific for AnxA2 (red), ADGRL2 (green), and σC (green). Enlarged images correspond to the region indicated by the white box in the merged image. The representative images are from three independent experiments. Scale bars, 20 µm.

Figure S2

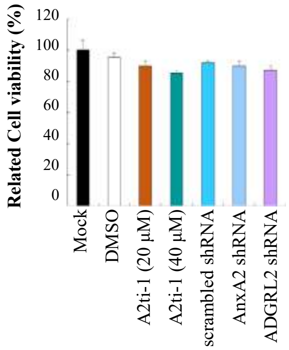

Supplementary Fig. S2. To investigate whether A2ti-1 inhibitor and shRNAs have deleterious effects on the cell, cell viability was assessed by MTT assay.

Figure S3

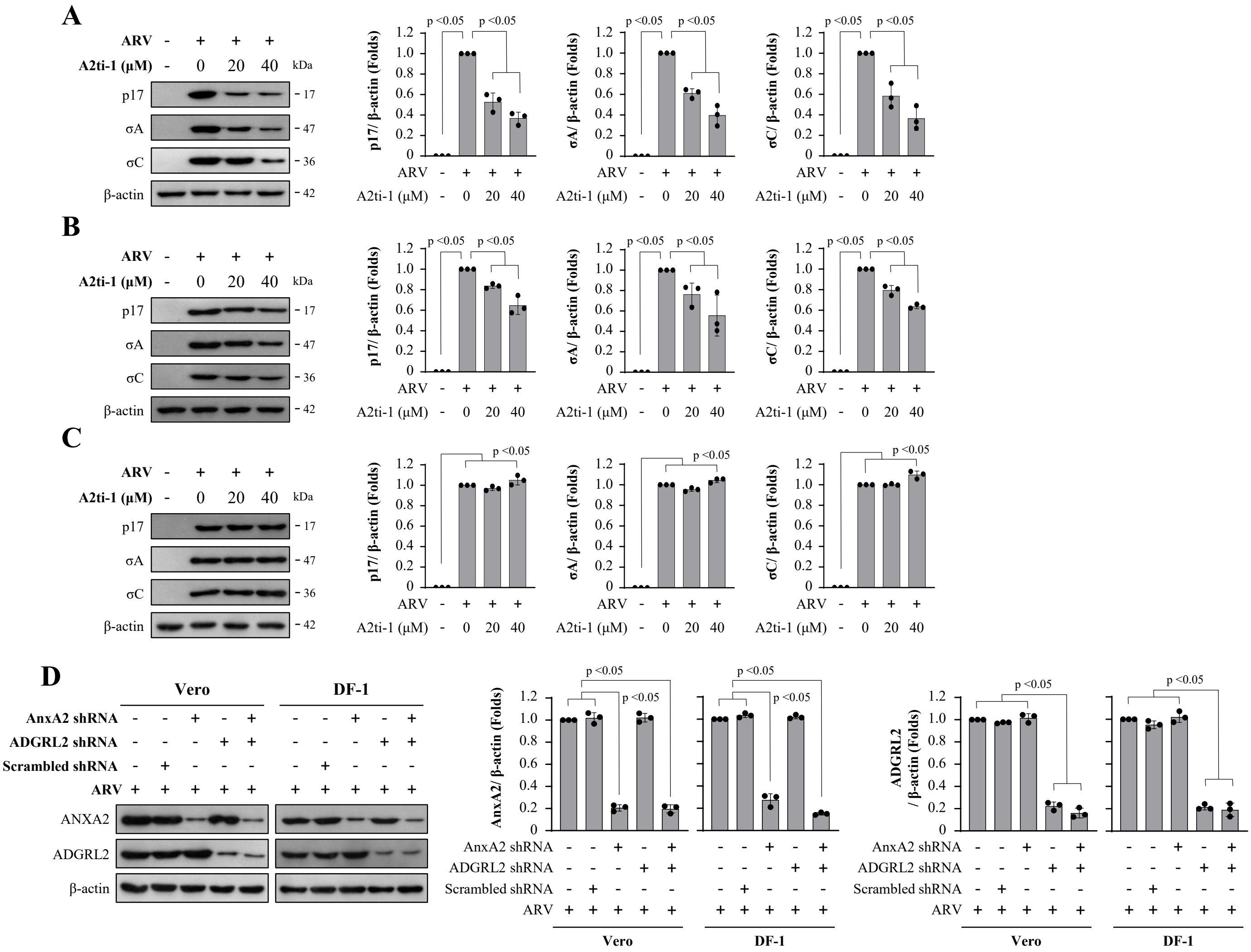

Supplementary Fig. S3. Inhibition of cell-surface AnxA2 and ADGRL2 suppressed expression of viral proteins. (A-C) Vero cells were pretreated with inhibitor A2ti-1 before (A), during (B), and after (C) infection for 2 hours. Cells were washed to remove the drug and further incubated with ARV at an MOI of 10 until 24 hours. The expression levels of viral proteins p17, σA, and σC were analyzed by Western blotting with the respective antibodies. (D) Since ADGRL2 inhibitor is not available, we used a shRNA to deplete the ADGRL2 gene. Cells were transfected with the indicated shRNAs for 6 h followed by infection with an MOI of 10 for 24 h. The protein levels were normalized to that for β-actin. The levels of the indicated proteins in the mock treatment were considered 1-fold. All experiments were performed in triplicate, and data are presented as the mean mean±SE. An unpaired two-tailed Student's t-test was performed for between-group comparisons using GraphPad Prism software version 8. The image shown is from a single experiment that is representative of at least three separate experiments.

Figure S4

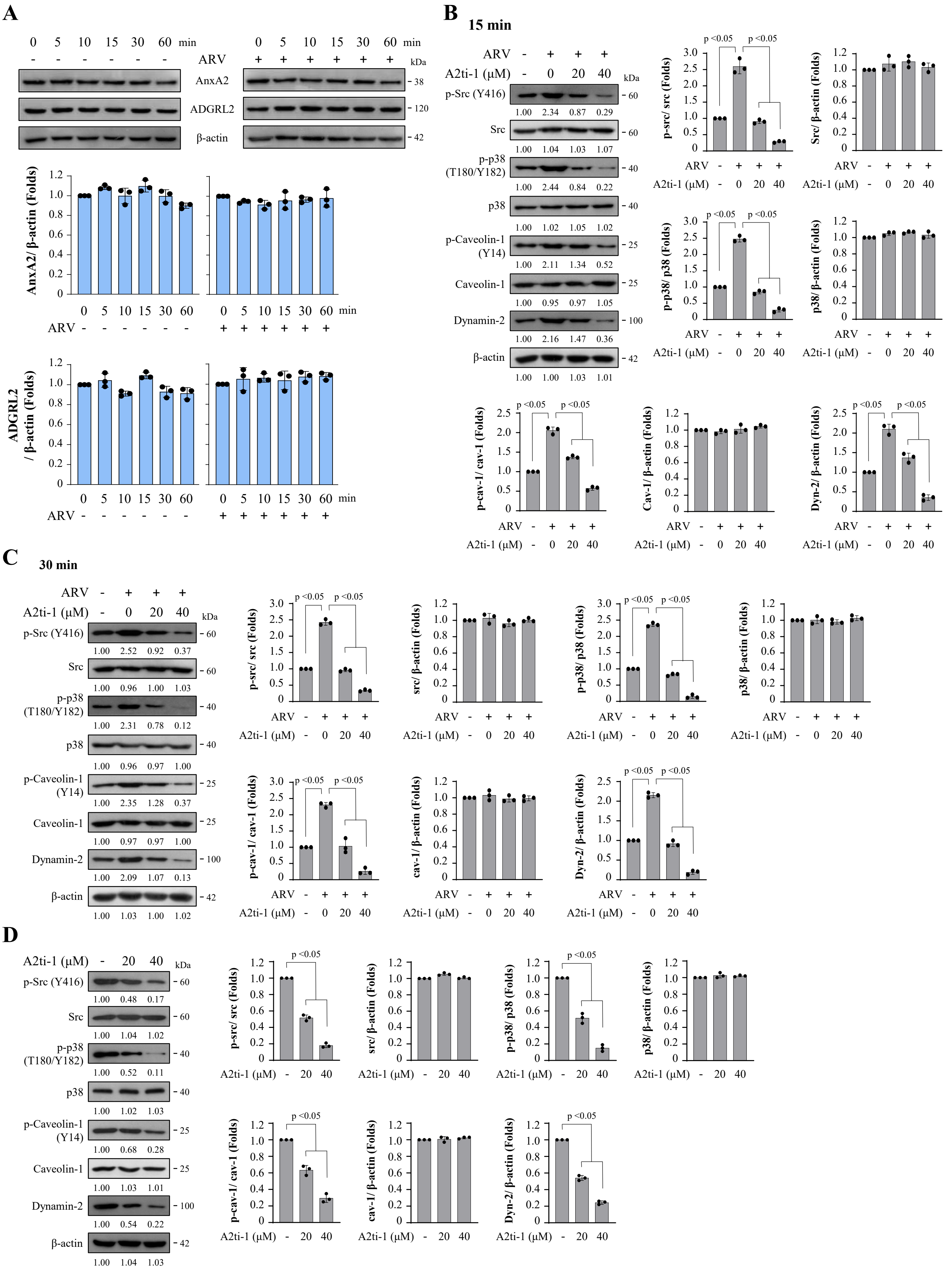
A
0 5 10 15 30 60 min
ARV
AnxA2
ADGRL2
β-actin
kDa
38
120
42
AnxA2/ β-actin (Folds)
ADGRL2 / β-actin (Folds)
B
15 min
ARV - + + +
A2ti-1 (μM) - 0 20 40
kDa
p-Src (Y416)
1.00 2.34 0.87 0.29
Src
1.00 1.04 1.03 1.07
p-p38 (T180/Y182)
1.00 2.44 0.84 0.22
p38
1.00 1.02 1.05 1.02
p-Caveolin-1 (Y14)
1.00 2.11 1.34 0.52
Caveolin-1
1.00 0.95 0.97 1.05
Dynamin-2
1.00 2.16 1.47 0.36
β-actin
1.00 1.00 1.03 1.01
p <0.05
p-src/ src (Folds)
Src/ β-actin (Folds)
p-p38/ p38 (Folds)
p38/ β-actin (Folds)
p-cav-1/ cav-1 (Folds)
Cav-1/ β-actin (Folds)
Dyn-2/ β-actin (Folds)
C
30 min
ARV - + + +
A2ti-1 (μM) - 0 20 40
kDa
p-Src (Y416)
1.00 2.52 0.92 0.37
Src
1.00 0.96 1.00 1.03
p-p38 (T180/Y182)
1.00 2.31 0.78 0.12
p38
1.00 0.96 0.97 1.00
p-Caveolin-1 (Y14)
1.00 2.35 1.28 0.37
Caveolin-1
1.00 0.97 0.97 1.00
Dynamin-2
1.00 2.09 1.07 0.13
β-actin
1.00 1.03 1.00 1.02
p <0.05
p-src/ src (Folds)
src/ β-actin (Folds)
p-p38/ p38 (Folds)
p38/ β-actin (Folds)
p-cav-1/ cav-1 (Folds)
cav-1/ β-actin (Folds)
Dyn-2/ β-actin (Folds)
D
A2ti-1 (μM) - 20 40
kDa
p-Src (Y416)
1.00 0.48 0.17
Src
1.00 1.04 1.02
p-p38 (T180/Y182)
1.00 0.52 0.11
p38
1.00 1.02 1.03
p-Caveolin-1 (Y14)
1.00 0.68 0.28
Caveolin-1
1.00 1.03 1.01
Dynamin-2
1.00 0.54 0.22
β-actin
1.00 1.04 1.03
p <0.05
p-src/ src (Folds)
src/ β-actin (Folds)
p-p38/ p38 (Folds)
p38/ β-actin (Folds)
p-cav-1/ cav-1 (Folds)
cav-1/ β-actin (Folds)
Dyn-2/ β-actin (Folds)

E

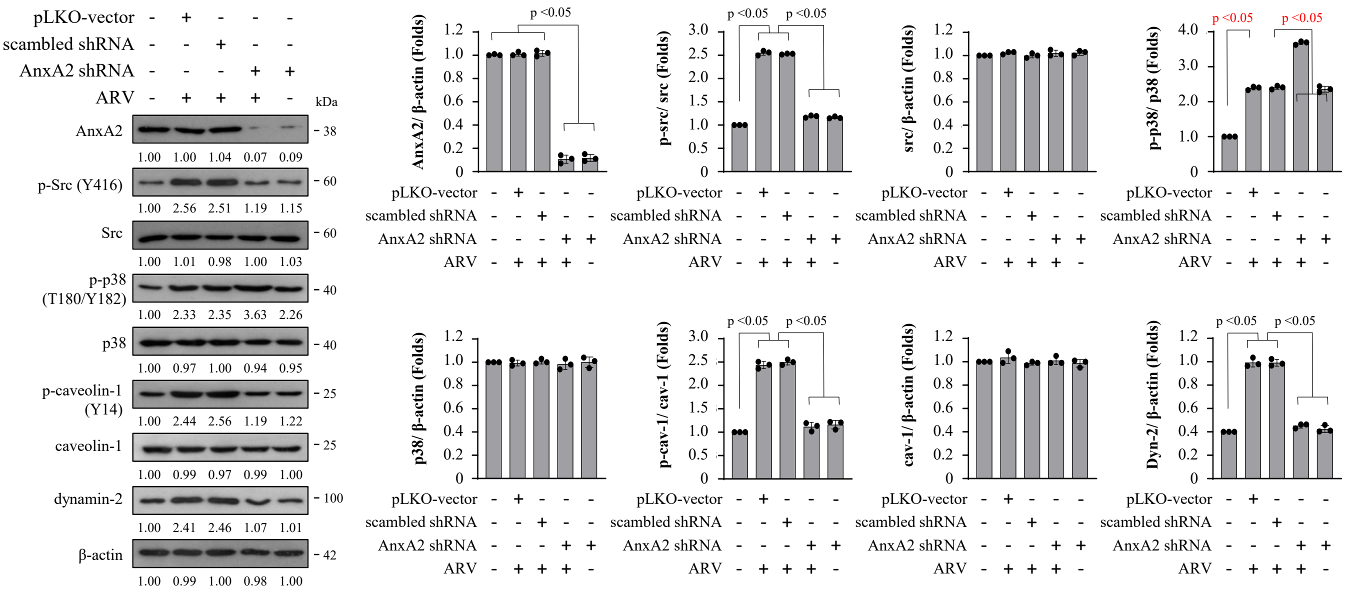

F

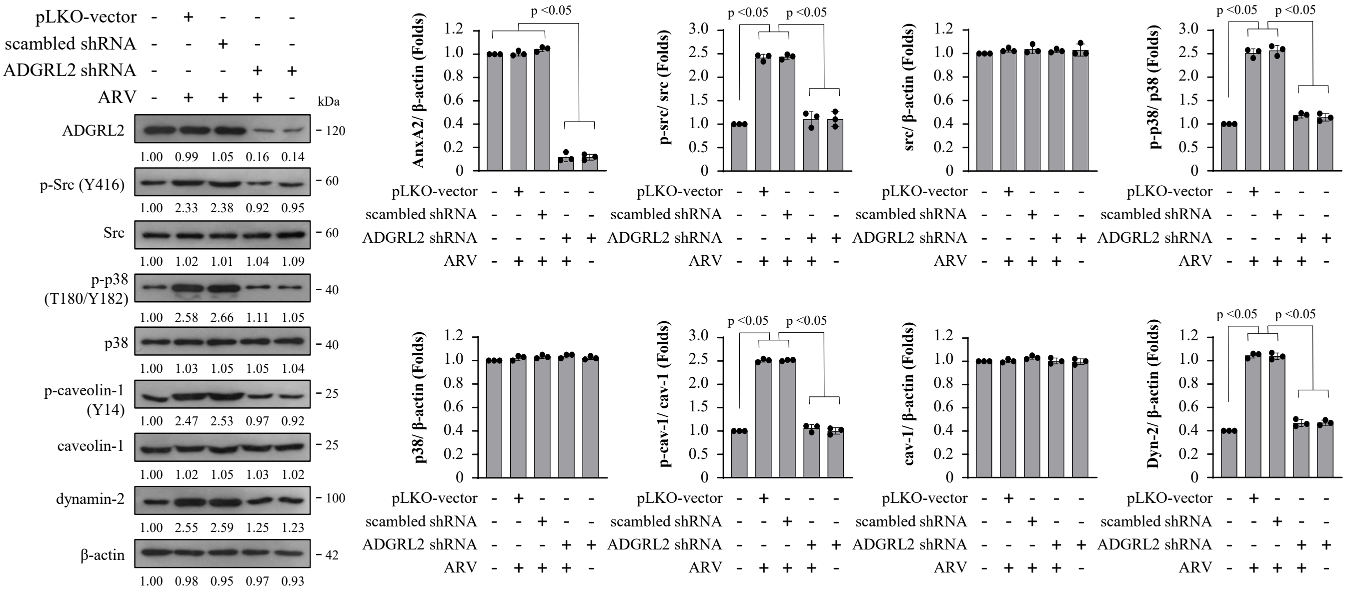

Supplementary Fig. S4. ARV interaction with the cell-surface AnxA2 activates Src and p38, which enhance expression levels of cavolin-1 and dynamin 2. (A) The levels of AnxA2 and ADGRL2 were examined in ARV-infected Vero cells at the indicated time points. (B-C) Vero cells were pretreated with inhibitor A2ti-1 for 1 hour followed by infection with ARV at an MOI of 10 for the indicated time points (15 and 30 min). (D) Vero cells were treated with inhibitor A2ti-1 for 1.5 hour without ARV. (E-F) Vero cells were transfected with the indicated shRNAs for 24 hours followed infection, with ARV at an MOI of 10 for 30 mins. Cell lysates were collected and immunoblotted with the respective antibody. The expression levels of the indicated proteins were analyzed by Western blotting with the respective antibodies and quantitated by densitometric analysis using ImageJ, normalized to β-actin. The levels of indicated proteins in the mock group was considered 1-fold. The predicted size of each protein was labeled at the right of gels and blots in kDa in each figure. All experiments were performed in triplicate, and data are presented as the mean± SE. An unpaired two-tailed Student's t-test was performed for between-group comparisons using GraphPad Prism software version 8. The image shown is from a single experiment that is representative of at least three separate experiments.

Figure S5

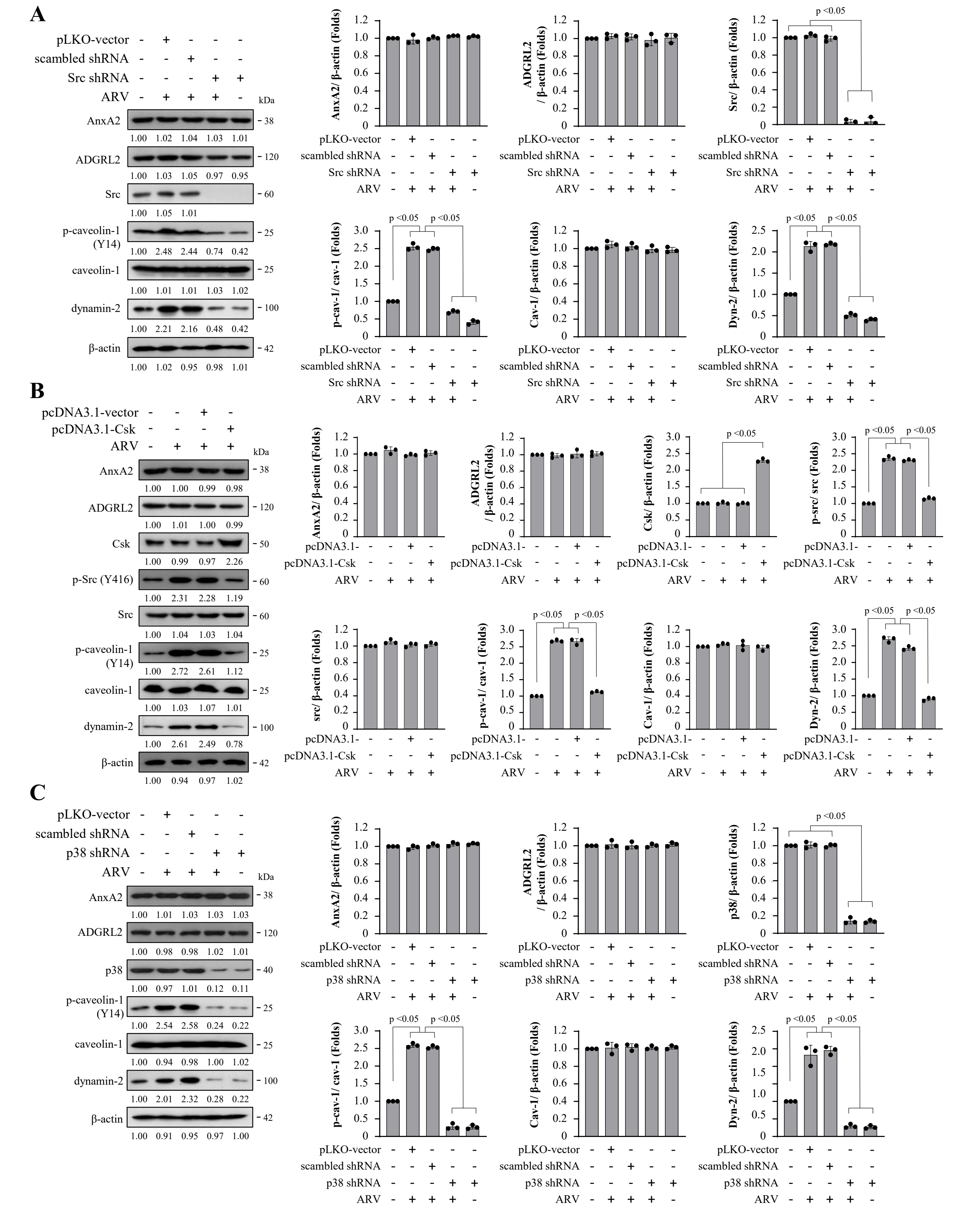

Supplementary Fig. S5. Src and p38MAPK play an important role in regulating expression levels of cavolin-1 and dynamin 2. (A-C) Vero cells were transfected with pcDNA3.1-Csk plasmid and different shRNAs for 6 hours followed by infection with ARV at an MOI of 10 for 24 hours. Cell lysates were collected at 24 hours post-infection and immunoblotted with respective antibodies. The protein levels were normalized to that for β-actin. The levels of the indicated proteins in the mock treatment were considered 1-fold. All experiments were performed in triplicate, and data are presented as the mean± SE. An unpaired two-tailed Student's t-test was performed for between-group comparisons using GraphPad Prism software version 8. The image shown is from a single experiment that is representative of at least three separate experiments.

Figure S6

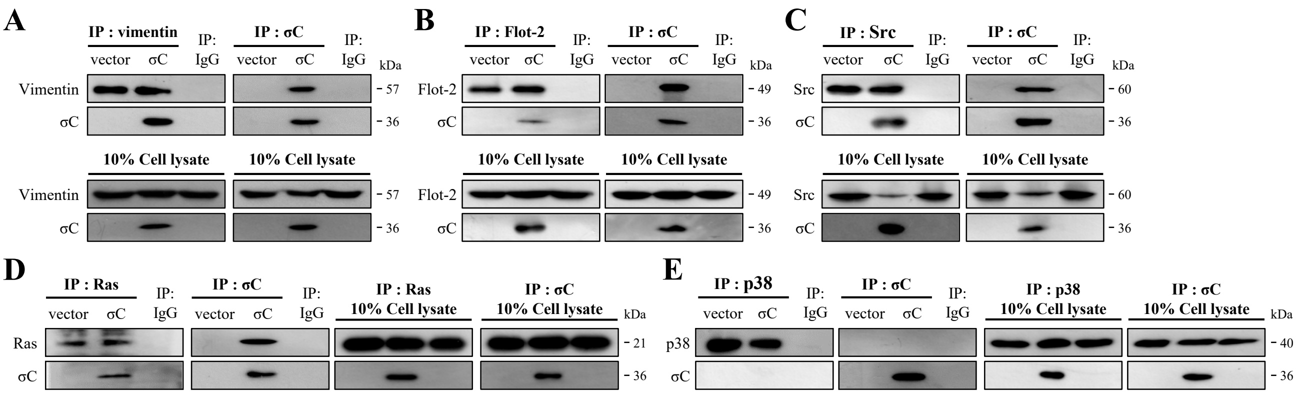

Supplementary Fig. S6. ARV σC interacts with cellular factors vimentin, Flot-2, Src, Ras, and p38MAPK. (A-E) In co-immunoprecipitation experiments, Vero cells were transfected with the pCI-neo-σC vector for 24 h. Cells lysates were immunoprecipitated with the indicated antibodies. The immunoprecipitated proteins were detected with the indicated antibodies by Western blot assay. Rabbit IgG served as negative control. All experiments were conducted in three independent experiments.

Figure S7

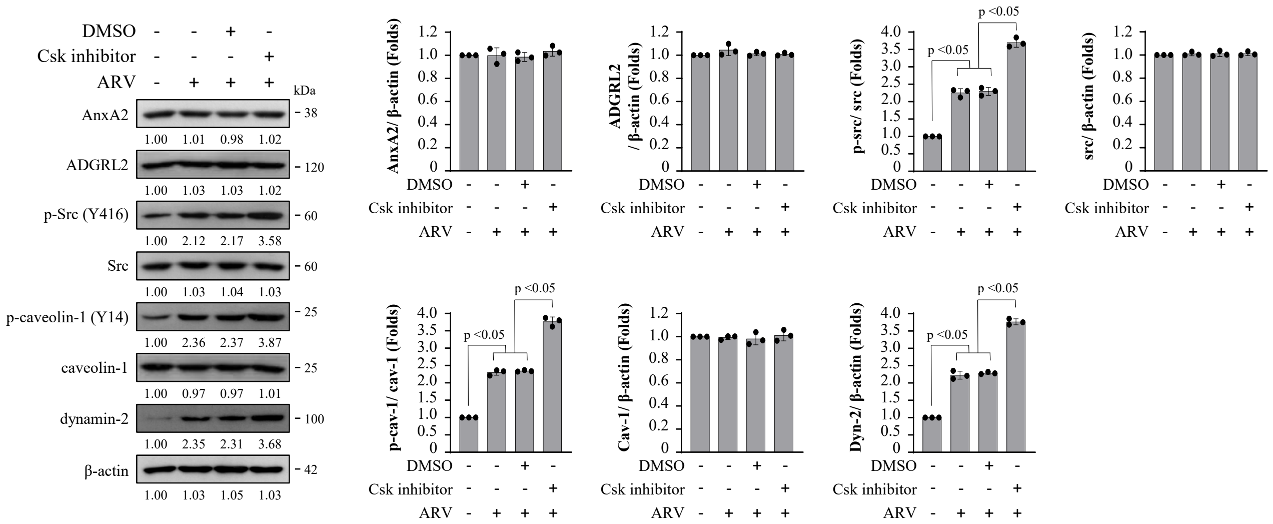

Supplementary Fig. S7. ARV reduces Cbp-Csk interaction at the early stage of life cycle. Vero cells were pretreated with the Csk inhibitor before infection for 2 hours. Cells were washed to remove the drug and further infected with ARV at an MOI of 10 for 24 hours. The expression levels of the indicated proteins were analyzed by Western blotting with the respective antibodies. The protein levels were normalized to that for β-actin. The levels of the indicated proteins in the mock treatment were considered 1-fold. All experiments were performed in triplicate, and data are presented as the mean± SE. An unpaired two-tailed Student's t-test was performed for between-group comparisons using GraphPad Prism software version 8. The image shown is from a single experiment that is representative of at least three separate experiments.

Figure S8

Figure 1A

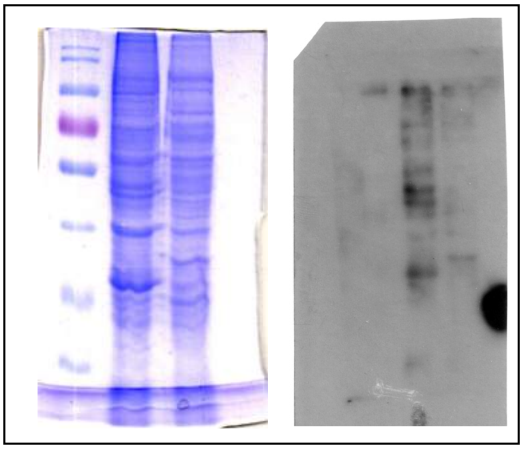

Figure 2A

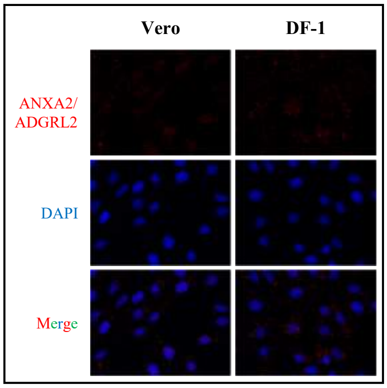

Figure 1B

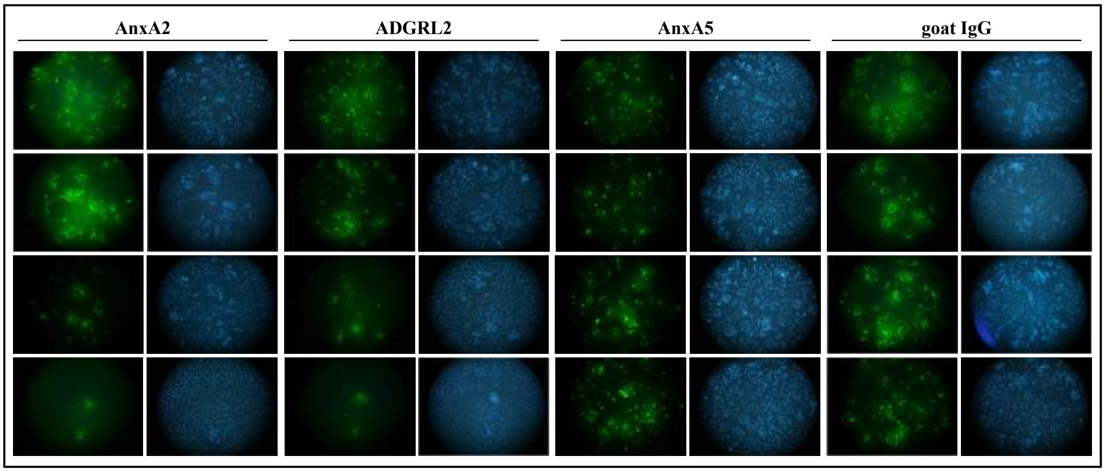

Figure 2B

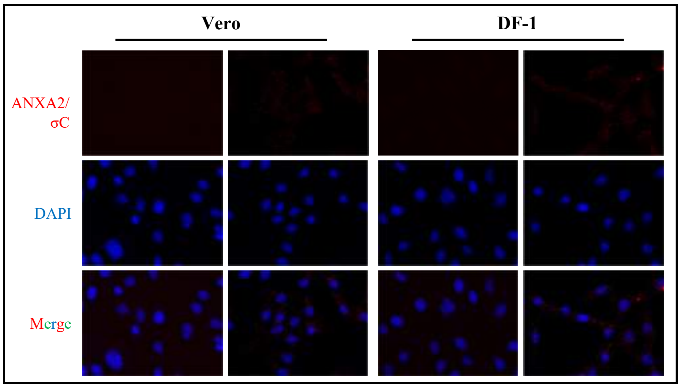

Figure 2C

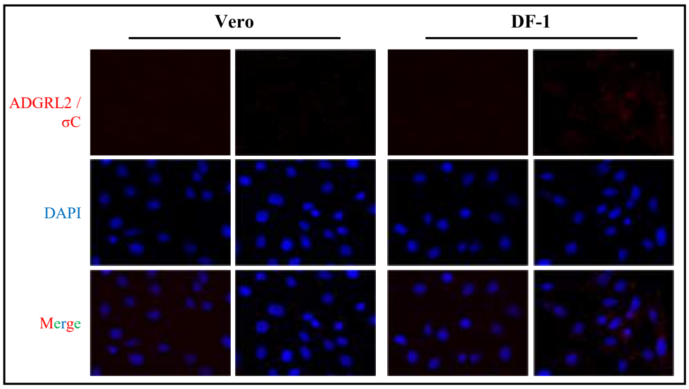

Figure 2E

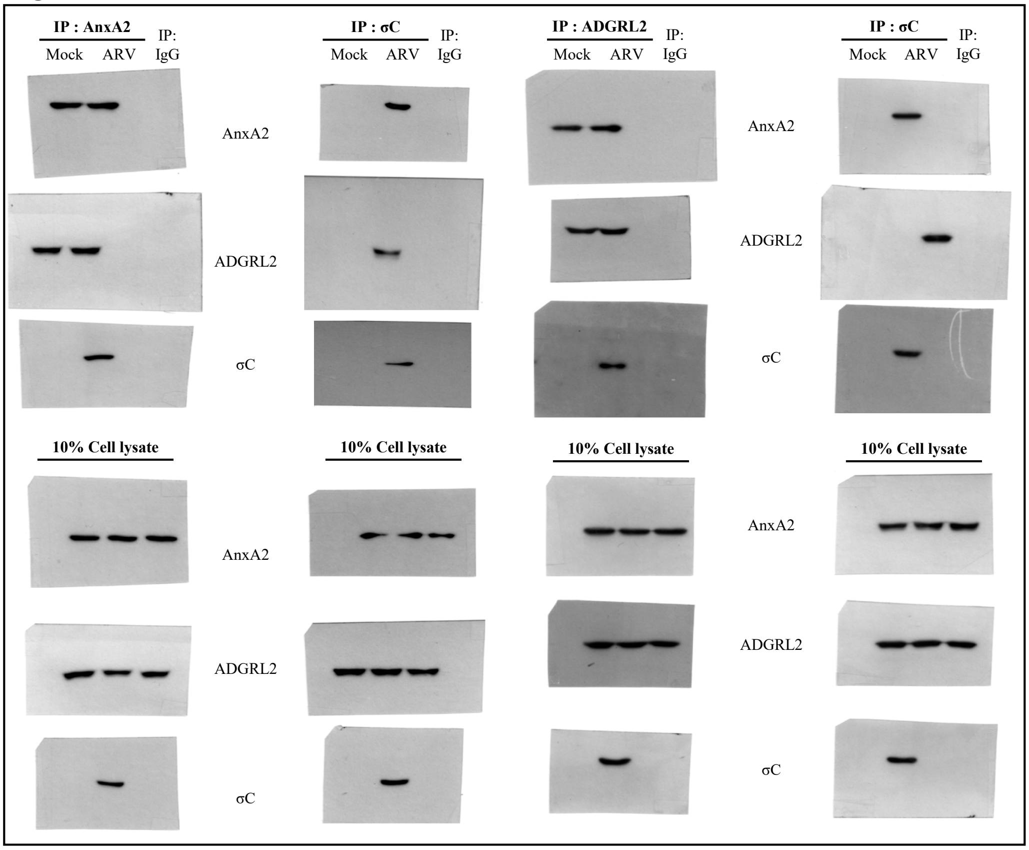

Figure 3B

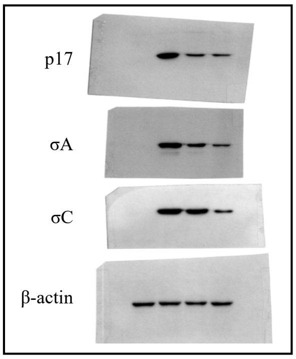

Figure 3C

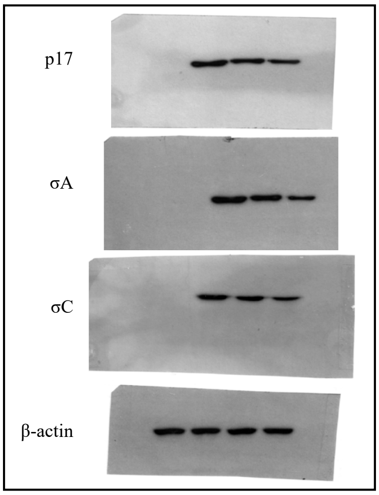

Figure 3D

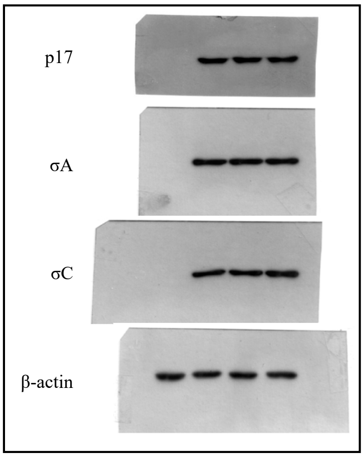

Figure 3E

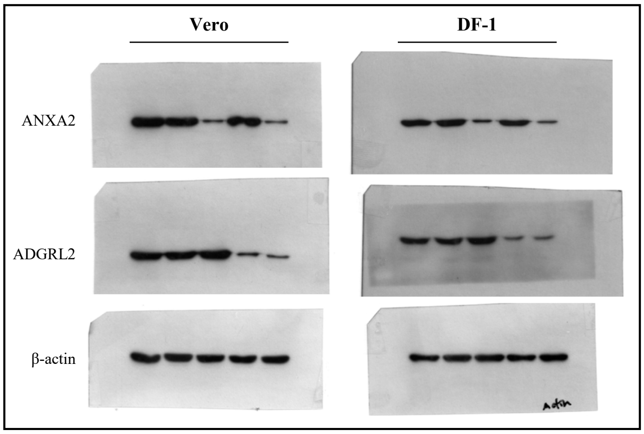

Figure 4A

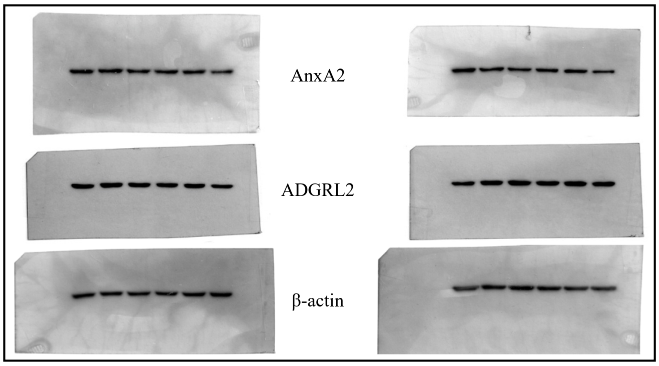

Figure 4B

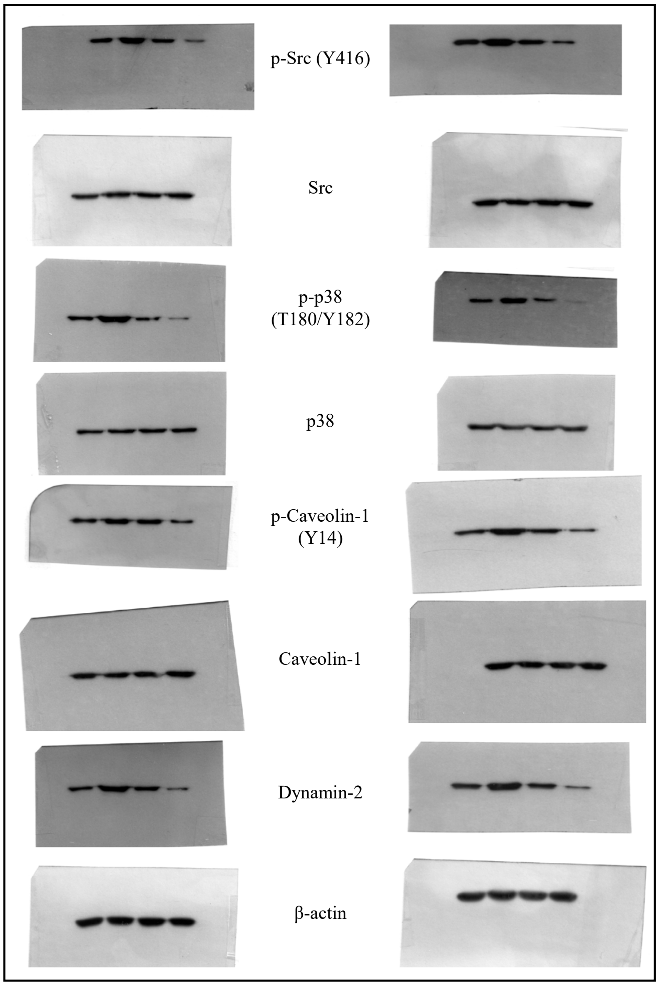

Figure 4C

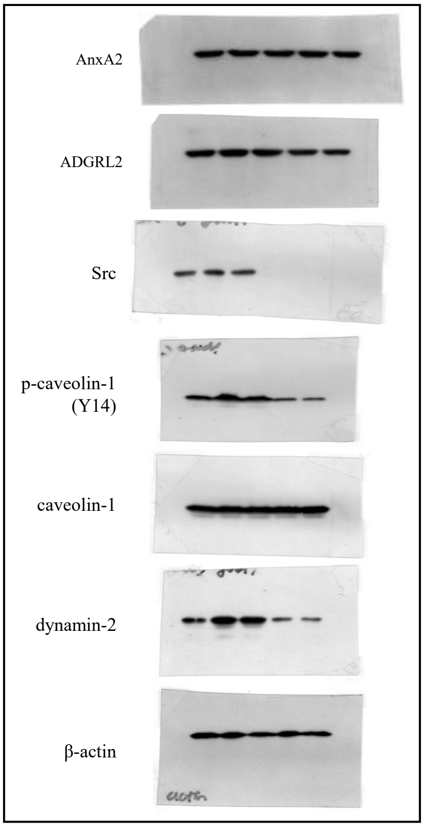

Figure 4D

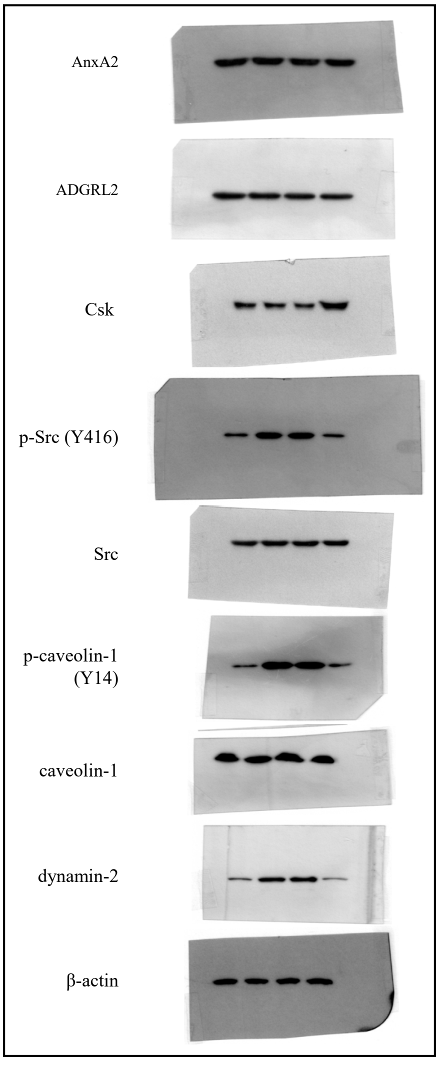

Figure 4E

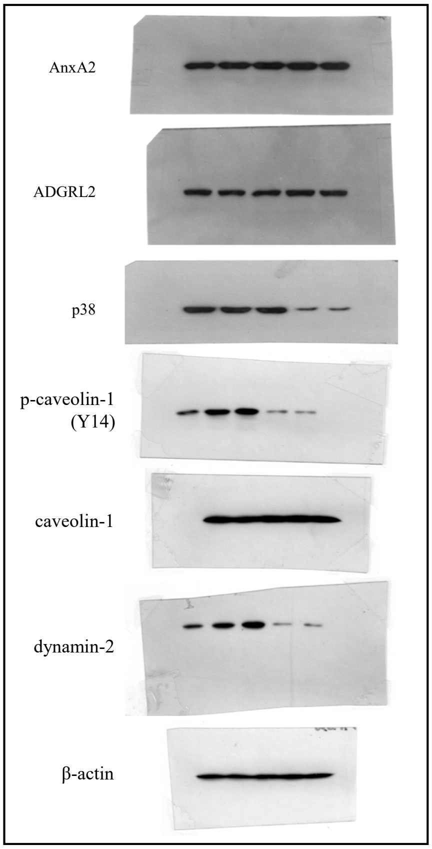

Figure 5B

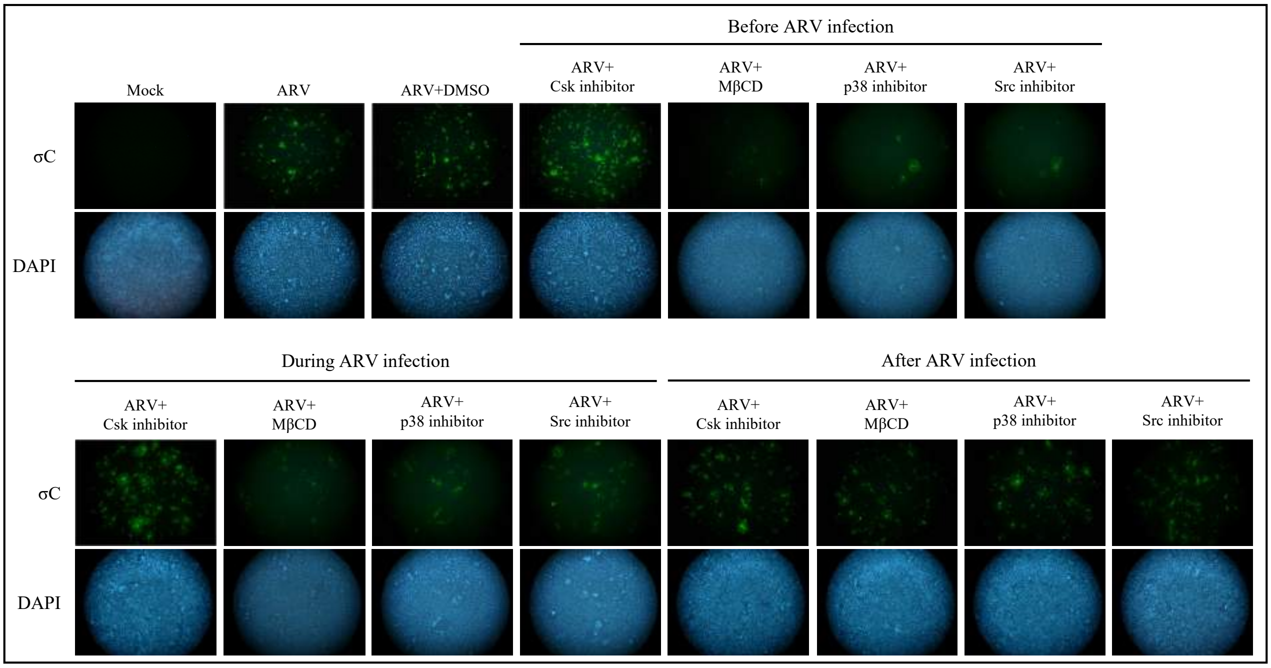

Figure 6A

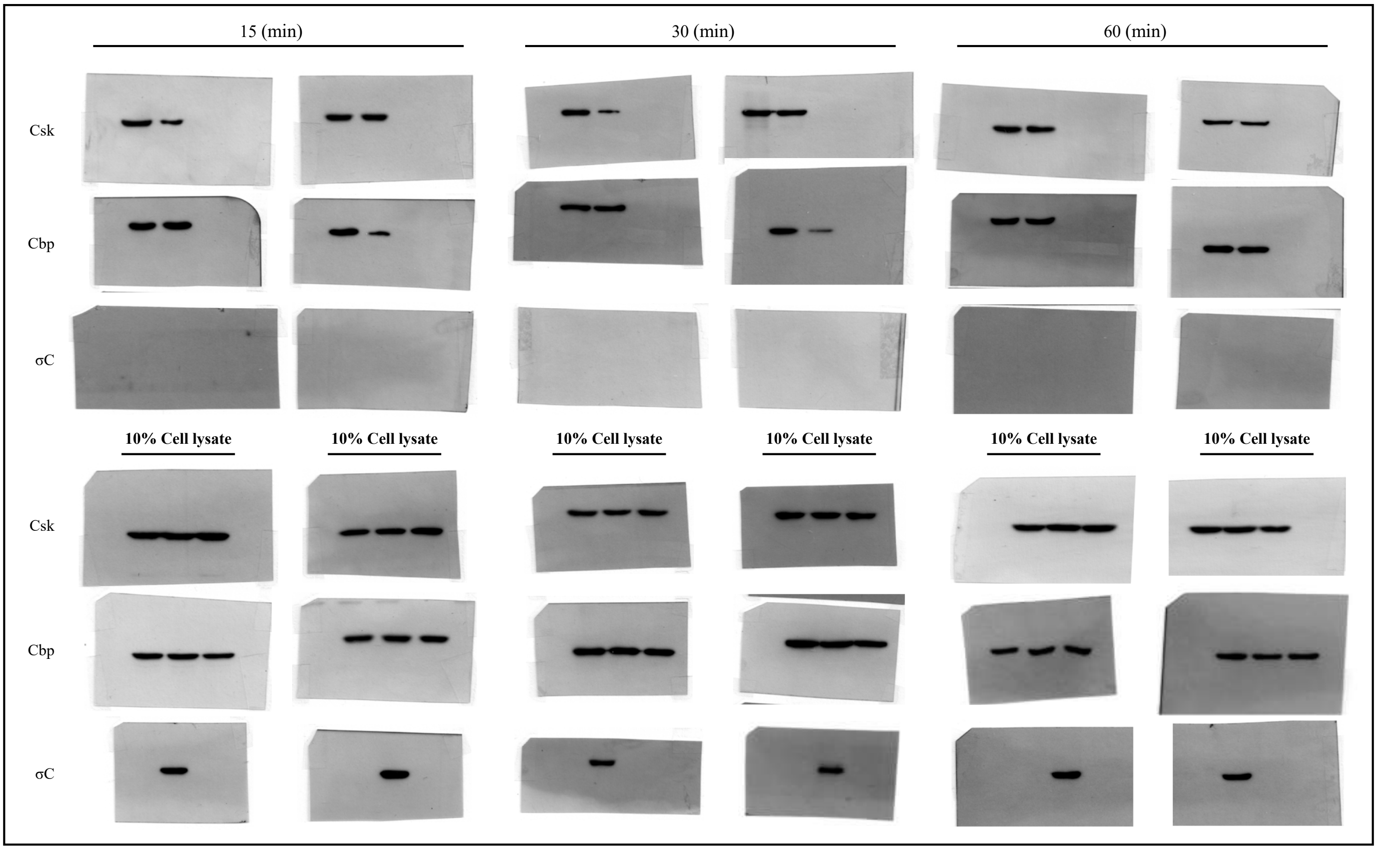

Figure 6C

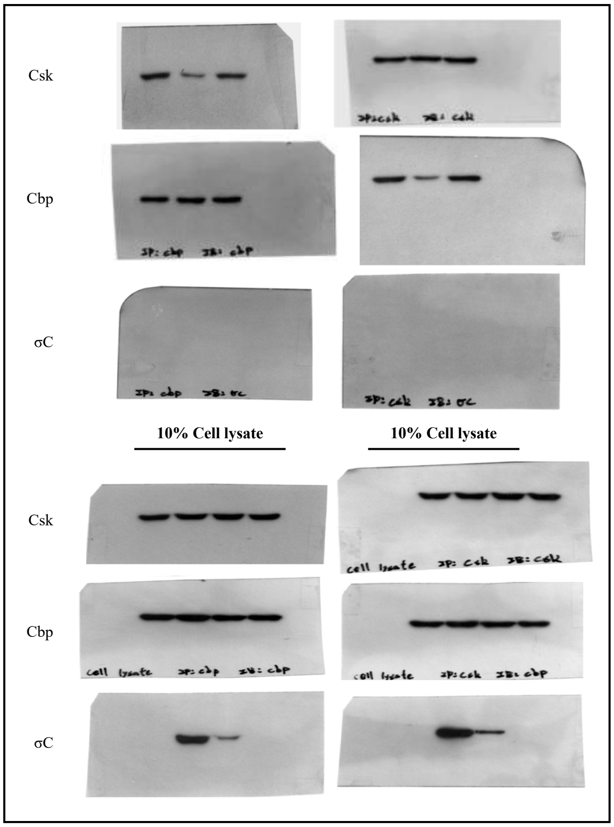

Figure 6D

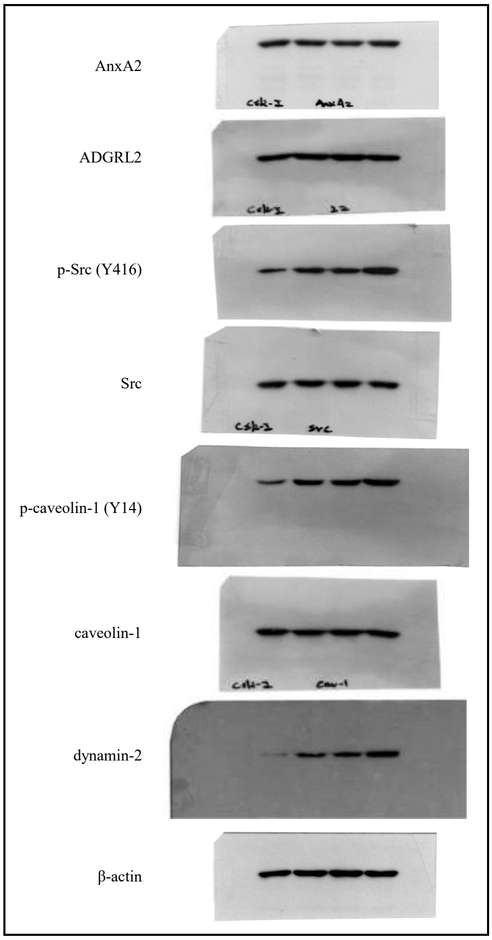

Figure S1A

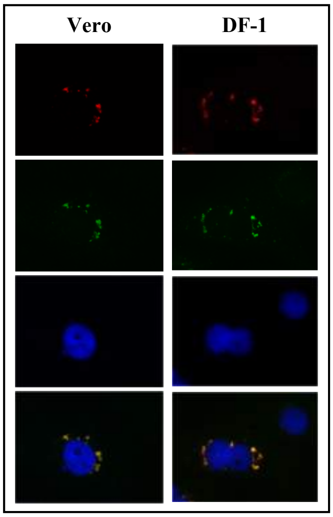

Figure S1B

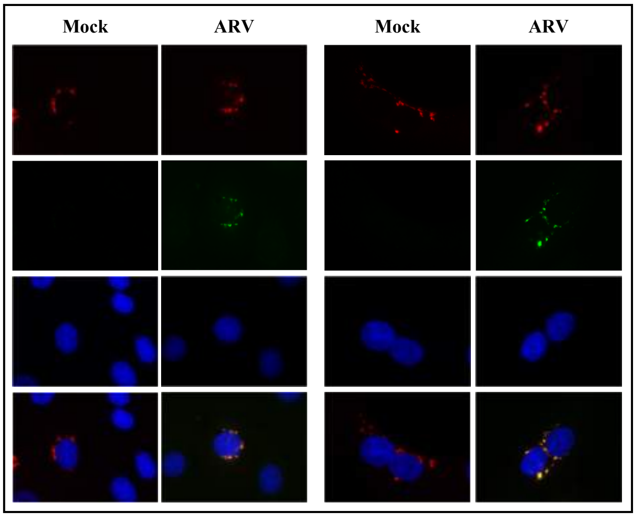

Figure S1C

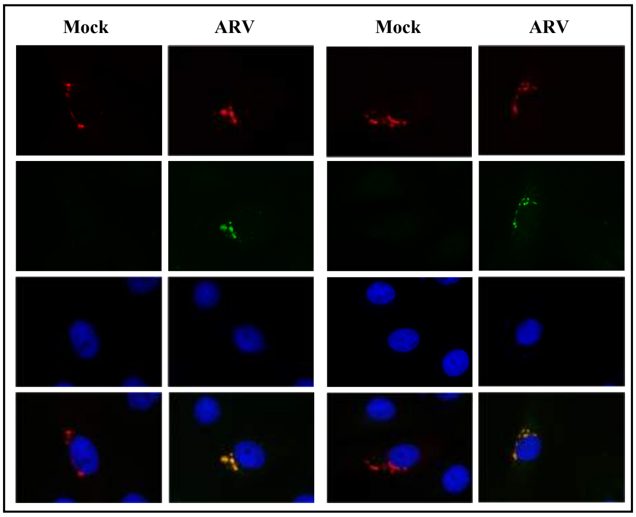

Figure S4D

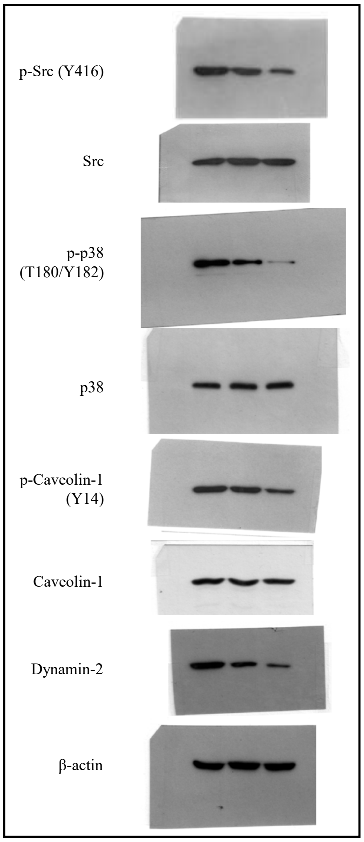

Figure S4E

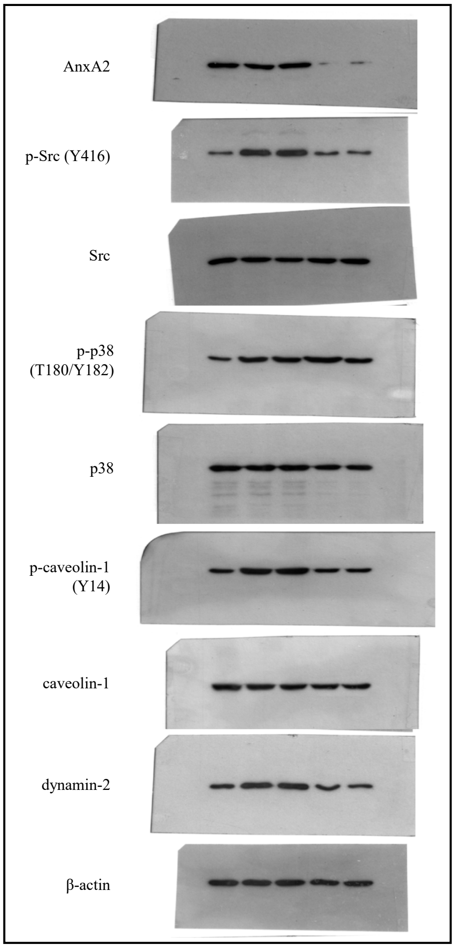

Figure S4F

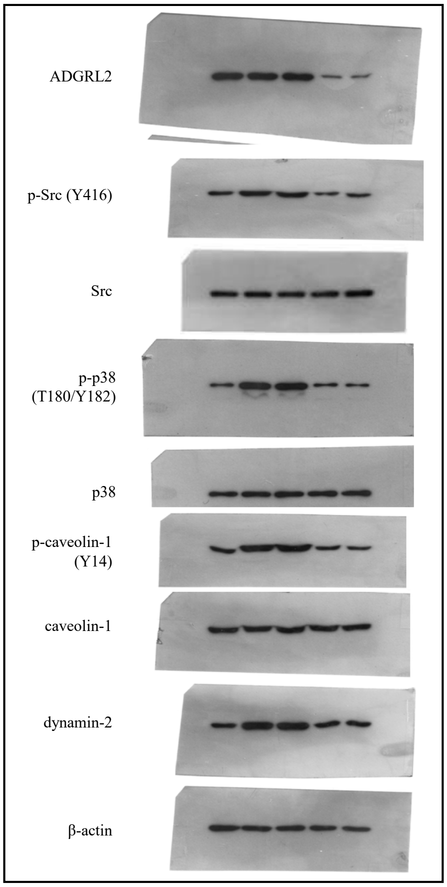

Figure S5A

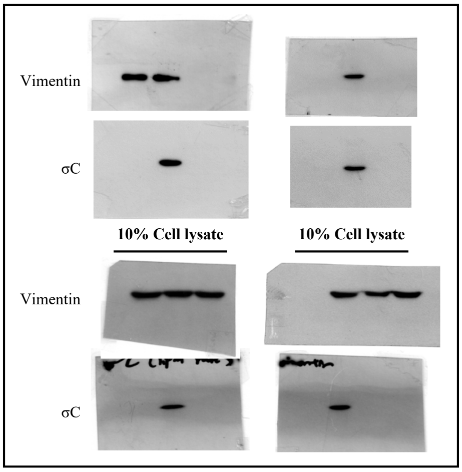

Figure S5B

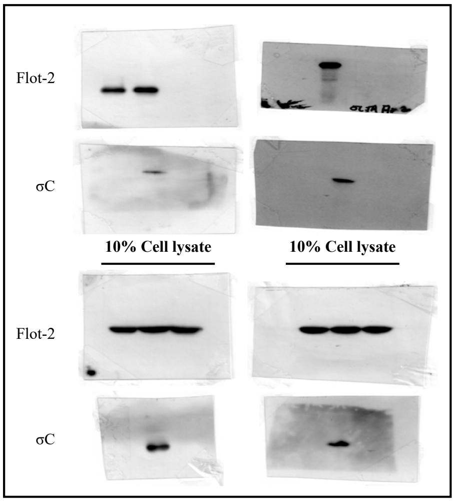

Figure S5C

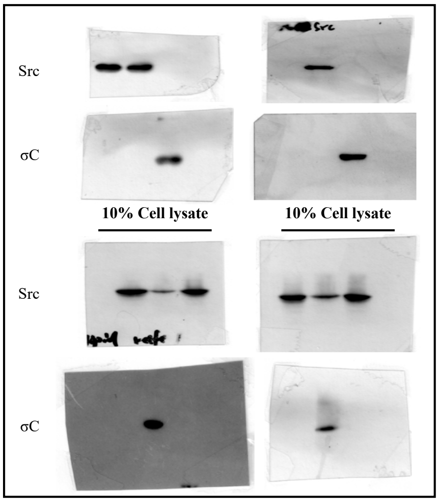

Figure S5D

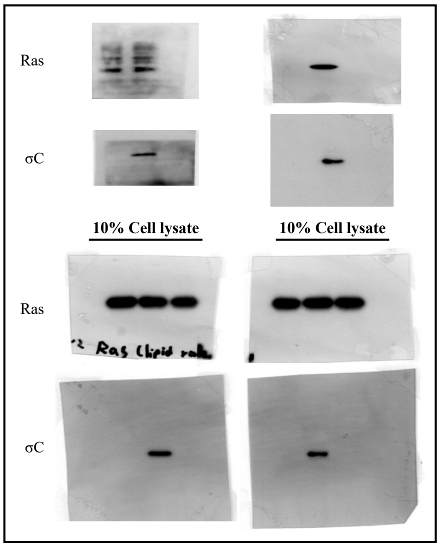

Figure S5E

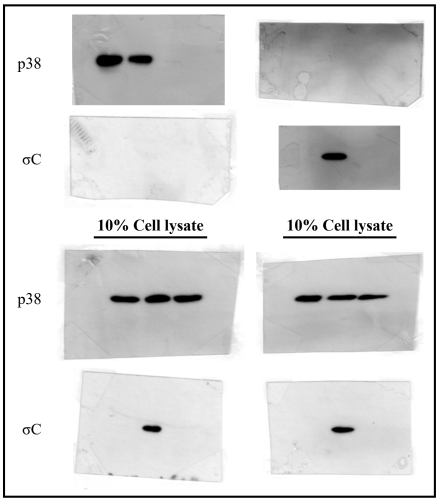

Supplementary Fig. S8. All original/uncropped blots or images.
